# Characterization of two key cytochrome P450 enzymes in *Isodon amethystoides* and *de novo* production of 3α,15β-dihydroxy-*ent-*kaurene in yeast

**DOI:** 10.1016/j.synbio.2026.08.004

**Published:** 2026-08-27

**Authors:** Yanying Chen, Xinqi Song, Yanan Wang, Yang Han, Meng Xia, Shijun Yuan, Ying Ma, Jian Wang, Qing Ma, Shuang Liu, Ping Su, Luqi Huang

**Affiliations:** a School of Pharmacy, Chengdu University of Traditional Chinese Medicine, Chengdu, 611137, China; b State Key Laboratory for Quality Ensurance and Sustainable Use of Dao-di Herbs, National Resource Center for Chinese Materia Medica, China Academy of Chinese Medical Sciences, Beijing, 100700, China; c Experimental Research Center, China Academy of Chinese Medical Sciences, Beijing, 100700, China

**Keywords:** *Isodon amethystoides*, Cytochrome P450 enzyme, Glaucocalyxin A, 3α,15β-dihydroxy-*ent-*kaurene, *de novo* production

## Abstract

Glaucocalyxin A (GLA), a well-known *ent-*kaurene diterpenoid, has emerged as a high-value bioactive natural product in pharmaceutical research owing to its potent and broad-spectrum activities. To date, its total synthesis has proven extremely challenging due to poor yield and intricate routes, and the biosynthetic pathway of GLA remains poorly understood. Herein, we identified two enzymes, IamCYP71D761 and IamCYP706V18, in *Isodon amethystoides*, which specifically mediate the C3α- and C15β-hydroxylation of *ent-*kaurene with high stereoselectivity and regioselectivity, respectively, generating 3α,15β-dihydroxy-*ent-*kaurene (**3**), a key precursor in the biosynthesis of GLA. In addition, we established an efficient *de novo* biosynthesis platform for **3** in *Saccharomyces cerevisiae*. Briefly, high-efficiency isozyme screening, protein engineering, increased acetyl-CoA synthesis, and copy number enhancement were applied to the *ent-*kaurene biosynthesis module. Subsequently, promoter optimization, competitive pathway knockout, and electron transfer optimization were introduced, resulting in production of **3** to 24.0 ± 0.2 mg/L in shake flask. In summary, this work highlights the pivotal roles of IamCYP71D761 and IamCYP706V18 in the heterologous biosynthesis of **3**, offering valuable insights for the further pathway reconstruction and *de novo* production of GLA, GLB, and other *ent-*kaurene diterpenoids.

## 1 Introduction

**Fig. 1:**
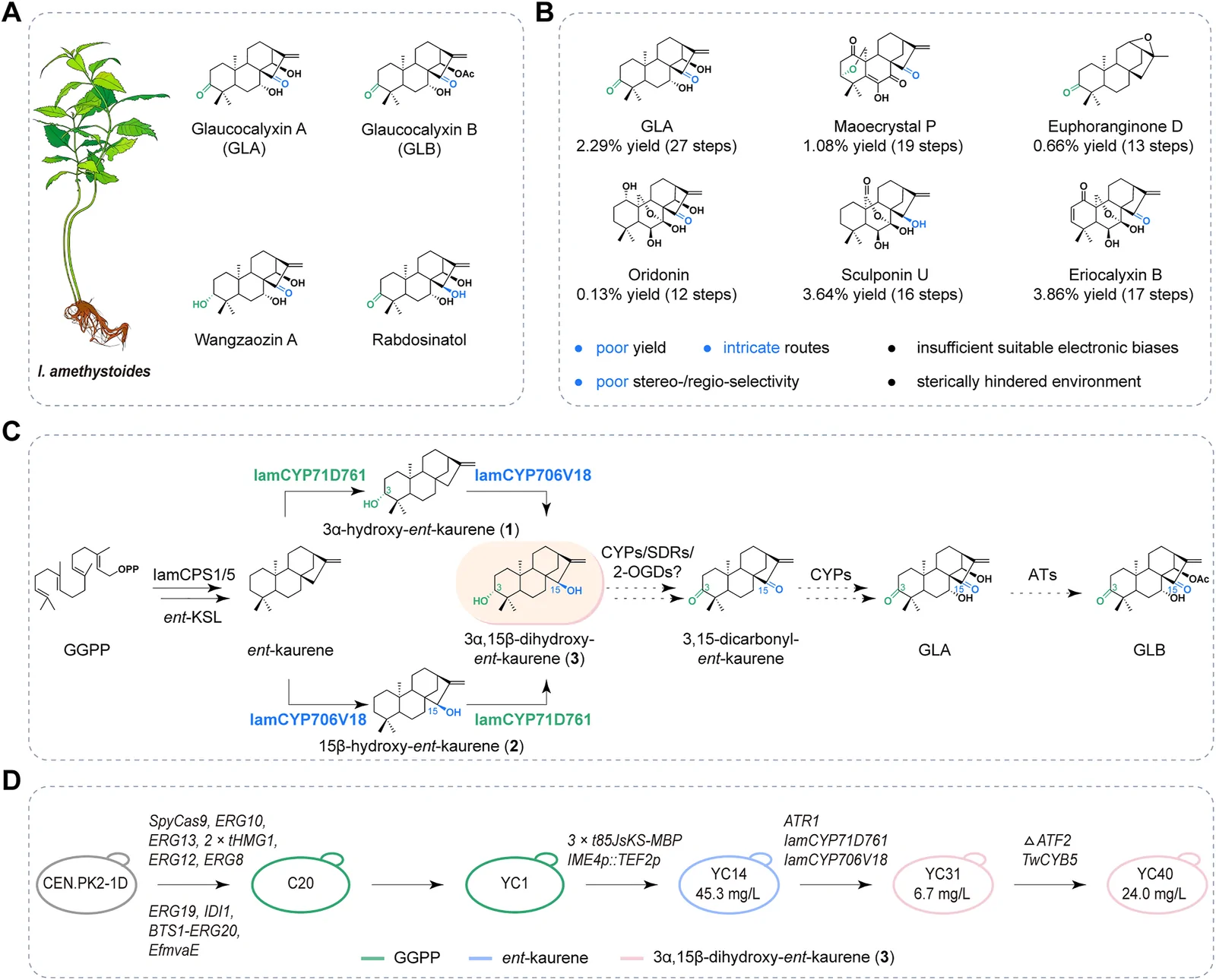
(A) Typical *ent-*kaurene diterpenoids in *I. amethystoides*. (B) Total synthesis of *ent-*kaurene diterpenoids. (C) Proposed biosynthetic pathway of GLA and GLB in *I. amethystoides*. (D) *De novo* production of **3** in engineered yeast strains.

*Ent-*kaurene diterpenoids are signature bioactive constituents of the *Isodon* genus, characterized by a unique [6,6,6,5]-fused tetracyclic skeleton and dense adjacent stereocenters. Owing to their remarkable pharmacological activities, many members are widely recognized as promising anticancer drug candidates [1]. As a representative species in the *Isodon* genus, *Isodon amethystoides* has been traditionally used in Chinese herbal medicine to treat various pulmonary disorders, including lobar pneumonia and tuberculosis [2,3]. Phytochemical studies have confirmed that glaucocalyxin A (GLA), glaucocalyxin B (GLB), wangzaozin A and rabdosinatol are typical *ent-*kaurene diterpenoids in *I. amethystoides* (Fig. 1A). GLA, a high-value bioactive natural product, has attracted extensive attention due to its diverse pharmacological properties. Pharmacological research has demonstrated that GLA markedly inhibits cell proliferation and triggers apoptosis in human hepatocellular carcinoma cells, while exhibiting prominent hepatoprotective effects against liver injury [4,5]. Moreover, GLA exhibits strong binding affinity to key molecular targets and possesses potent inhibitory activity against non-small cell lung cancer [6]. In comparison, GLB primarily functions by suppressing key enzymes associated with DNA replication and regulating telomere shortening in gastric cancer cells [7]. Wangzaozin A shows significant cytotoxicity against SGC-7901 gastric cancer cells by inhibiting cell proliferation and inducing apoptotic cell death [8].

Owing to the unique structural features and pharmacological properties, *ent-*kaurene diterpenoids have attracted sustained attention from synthetic chemists [9]. To date, numerous total syntheses of these compounds have been accomplished, including GLA, maoecrystal P, euphoranginone D, oridonin, sculponin U, and eriocalyxin B. Nevertheless, these synthetic routes generally require 12–27 steps with low yields of only 0.13%–3.86%, primarily due to poor stereoselectivity and regioselectivity, insufficient suitable electronic biases, sterically hindered environment (Fig. 1B, Fig. S1A) [[10], [11], [12], [13], [14], [15]]. In contrast, synthetic biology has emerged as a promising strategy for producing *ent-*kaurene diterpenoids [16]. Current evidence demonstrates that diterpenoid synthases (diTPSs) facilitate the sequential cyclization of (*E*,*E*,*E*)-geranylgeranyl diphosphate (GGPP) to generate the *ent-*kaurene. Specifically, *Ent-*copalyl diphosphate synthase (*ent-*CPS, class II diTPS) typically catalyzes the cyclization of GGPP into *ent*-copalyl diphosphate (*ent-*CPP), which is subsequently catalyzed by *ent-*kaurene synthase-like terpene synthase (*ent-*KSL, class I diTPS) to produce *ent*-kaurene. The carbon skeleton of *ent*-kaurene is further subjected to structural modifications by diverse enzymes, including cytochrome P450 (CYP) enzymes, short-chain dehydrogenase/reductases (SDRs), 2–oxoglutarate-dependent dioxygenase (2–OGDs) and acetyltransferases (ATs), to yield GLA and GLB [[17], [18], [19], [20], [21], [22]]. Although several plant-derived CYPs are known to mediate selective hydroxylation of the *ent-*kaurene skeleton, the biosynthetic pathways responsible for GLA and GLB in *I. amethystoides* remain unelucidated (Fig. 1C, Fig. S1B) [[23], [24], [25], [26], [27], [28], [29], [30], [31]].

In this study, two CYP enzymes, IamCYP71D761 and IamCYP706V18, were identified from *I. amethystoides*. These enzymes acted as C3α- and C15β-hydroxylases with high regio- and stereoselectivity, yielding 3α-hydroxy-*ent-*kaurene (**1**), 15β-hydroxy-*ent-*kaurene (**2**), and 3α,15β-dihydroxy-*ent-*kaurene (**3**) (Fig. 1C). Starting with the GGPP-producing yeast strain YC1, we constructed the *ent-*kaurene-producing strain YC14 through isoenzyme screening, protein engineering (enzyme solubility optimization and N-terminal truncation), IME4 (yeast m^6^A methyltransferase)-mediated acetyl-CoA accumulation, and gene copy number amplification, yielding 45.3 ± 3.0 mg/L *ent-*kaurene in shake flask. Subsequently, cytochrome P450 reductase 1 (ATR1) from *Arabidopsis thaliana*, IamCYP71D761, and IamCYP706V18 were heterologously expressed, combined with promoter engineering, competitive pathway knockout, and electron transfer optimization. The final engineered strain YC40 achieved the first *de novo* biosynthesis of **3** from glucose, with a titer of 24.0 ± 0.2 mg/L in shake-flask fermentation (Fig. 1D). In conclusion, this study highlights IamCYP71D761 and IamCYP706V18 as key enzymes that facilitate the elucidation of GLA and GLB biosynthetic pathways in *I. amethystoides*, and lays a foundation for pathway reconstruction and efficient microbial biosynthesis of GLA, GLB, and other *ent-*kaurene diterpenoids.

## 2 Material and methods

### 2.1 Plant material, strains, plasmids, primers and reagents

Seedlings of *I. amethystoides* from Suzhou city, Anhui Province, China were cultivated for 8 weeks at 25 °C under a 16-h-light/8-h-dark photoperiod. The roots, stems, and leaves were harvested and stored at −80 °C for subsequent metabolic and transcriptomic analysis. Authentic GLA and GLB standards were purchased from Shanghai Yuanye Biotechnology Co., Ltd (Shanghai, China). Compound *ent*-kaurene was the gift from Prof. Xiao Zhang (Southwest University). All strains, plasmids, and primers used in this study are listed in Table 1, Table S1, and Table S2.

**Table 1:** *S. cerevisiae* strains used in this study.

Strain	Parent strain	Genotype	Source
CEN.PK2-1D	/	MATα; *ura3-52*; *trp1-289*; *leu2-3112*; his3Δ1; *MAL2-8C*; *SUC2*	*/*
C20	CEN.PK2-1D	XI-5:P_TEF1_-Cas9-T_CYC1_; Gal80Δ::P_GAL1_-ERG10-T_TPL1_-P_GAL10_-ERG13-T_PGI_; Ypl062wΔ::P_GAL1_-tHMG1-T_ADH1_-P_GAL10_-ERG12-T_PDC1_; XI-3:P_GAL1_-ERG8-T_RPS2_-P_GAL10_-ERG19-T_TDH1_; Rox1Δ::P_GAL1_-IDI1-T_CCW12_-P_GAL10_-BTS1-ERG20-T_RPL9A_; YPRCΔ15:P_GAL1_-tHMG1-T_ADH1_-P_GAL10_-EfmvaE-T_PDC1_	[29]
YC1	C20	XII-5::P_GAL1_-XdGGPPS-T_CYC1_	this study
YC2	YC1	XII-1::P_GAL1_-GfKS-T_CYC1_	this study
YC3	YC1	XII-1::P_GAL1_-JsKS-T_CYC1_	this study
YC4	YC1	XII-1::P_GAL1_-PspKS-T_CYC1_	this study
YC5	YC1	XII-1::P_GAL1_-t85JsKS-MBP-T_CYC1_	this study
YC6	YC1	XII-1::P_GAL1_-t126JsKS-MBP-T_CYC1_	this study
YC7	YC5	*IME4*p::*GAL1*p	this study
YC8	YC5	*IME4*p::*SptGAL2*p	this study
YC9	YC5	*IME4*p::*PGK1*p	this study
YC10	YC5	*IME4*p::*TEF2*p	this study
YC11	YC5	*IME4*p::*TDH3*p	this study
YC12	YC5	*IME4*p::*TDH3*p-RPS25Ai	this study
YC13	YC10	XII-4::P_TDH*3*_-t85JsKS-MBP-T_CYC1_	this study
YC14	YC13	308a::P_TDH3_-t85JsKS-MBP-T_CYC1_	this study
YC15	YC14	int14::P_GAL1_-ATR1-T_CYC1_	this study
YC16	YC15	P_GAL1_-IamCYP71D761-T_CYC1_	this study
YC17	YC15	P_SptGAL2_-IamCYP71D761-T_CYC1_	this study
YC18	YC15	P_TEF1_-IamCYP71D761-T_CYC1_	this study
YC19	YC15	P_TDH3_-IamCYP71D761-T_CYC1_	this study
YC20	YC15	P_TDH3-RPS25Ai_-IamCYP71D761-T_CYC1_	this study
YC21	YC15	P_eTDH3_-IamCYP71D761-T_CYC1_	this study
YC22	YC15	P_eTDH3-RPS25Ai_-IamCYP71D761-T_CYC1_	this study
YC23	YC15	1414a::P_eTDH3_-IamCYP71D761-T_CYC1_	this study
YC24	YC23	P_GAL1_-IamCYP706V18-T_CYC1_	this study
YC25	YC23	P_SptGAL2_-IamCYP706V18-T_CYC1_	this study
YC26	YC23	P_TEF1_-IamCYP706V18-T_CYC1_	this study
YC27	YC23	P_TDH3_-IamCYP706V18-T_CYC1_	this study
YC28	YC23	P_TDH3-RPS25Ai_-IamCYP706V18-T_CYC1_	this study
YC29	YC23	P_eTDH3_-IamCYP706V18-T_CYC1_	this study
YC30	YC23	P_eTDH3-RPS25Ai_-IamCYP706V18-T_CYC1_	this study
YC31	YC23	106a::P_eTDH3_-IamCYP706V18-T_CYC1_	this study
YC32	YC31	ΔATF1	this study
YC33	YC31	ΔATF2	this study
YC34	YC33	1012b::P_eTDH3_-IamCYP706V18-T_CYC1_	this study
YC35	YC38	XI-6::P_eTDH3_-IamCYP706V18-T_CYC1_	this study
YC36	YC33	XI-6::P_eTDH3_-IamCYP71D761-T_CYC1_	this study
YC37	YC33	X-2::P_GAL10_-AaCYB5-T_ADH1_	this study
YC38	YC33	X-2::P_GAL10_-CrCYB5-T_ADH1_	this study
YC39	YC33	X-2::P_GAL10_-RsCYB5-T_ADH1_	this study
YC40	YC33	X-2::P_GAL10_-TwCYB5-T_ADH1_	this study

### 2.2 Metabolite analysis of GLA and GLB in *I. amethystoides*

GLA and GLB standards were dissolved in methanol (Thermo Fisher, Germany) at serial concentrations of 1, 5, 10, 20, 50, and 80 μg/mL for standard-curve construction. Roots, stems, and leaves were ground in liquid nitrogen using a Retsch MM400 mixer mill (Retsch GmbH) and then stored at −80 °C for at least 4 h before freeze-drying for 48 h (Christ ALPHA 1-2, Germany). For metabolite extraction, 20 mg of the lyophilized tissue powder was mixed with 1 mL of methanol, sonicated at room temperature for 30 min, and then centrifuged. The resulting supernatant was collected for subsequent analysis. All extractions were performed with three biological replicates for each tissue type. Prior to ultra-high performance liquid chromatography (UPLC) analysis, the collected supernatant was filtered through a 0.22 μm polytetrafluoroethylene (PTFE) membrane filter, and the filtrate was injected into the UPLC system for detection.

UPLC analysis was performed using Waters ACQUITY UPLC™ I-Class system (Waters Corp., Milford, MA, USA) equipped with a Waters ACQUITY UPLC HSS T3 analytical column (2.1 mm × 100 mm, 1.8 μm). The flow rate was set at 0.4 mL·min^−1^, and the injection volume was 1 μL. The mobile phases consisted of 0.1% formic acid in water (solvent A) and acetonitrile (solvent B), the gradient program was as follows: 0–2 min, 70% A; 2–10 min, 70%–25% A; 10–15 min, 25%–5% A; 15–18 min, 5% A; 18–18.5 min, 5%–70% A; 18.5–21 min, 70% A. The contents of GLA and GLB in three tissues (roots, stems and leaves) were quantified by comparing the peak area with those of the corresponding standards using linear regression.

### 2.3 RNA isolation, cDNA synthesis, and RNA sequencing (RNA-seq)

Total RNA was extracted from the roots, stems and leaves of *I. amethystoides* using RNA extract kit (Huayueyang Biotechnology, Beijing, China) according to the manufacturer's protocols. The integrity and concentration of total RNA were assessed by agarose gel electrophoresis and the RNA Nano 6000 Assay Kit of the Bioanalyzer 2100 system (Agilent Technologies, CA, USA). For first-strand cDNA synthesis, 2 μg of high-quality total RNA was reverse-transcribed using the PrimeScript 1st Strand cDNA Synthesis Kit (Takara Bio, Beijing, China) according to the manufacturer's protocols. The cDNA library was sequenced on the Illumina Novaseq 6000 platform by Novogene Bioinformatics Technology Co. Ltd. RNA-seq analysis generated a total of 62,745 unigenes from the three tissues, with three biological replicates performed for each tissue sample.

### 2.4 Bioinformatics and phylogenetic tree analysis

The coding sequences of *IamCPS*s, *IamKSLs*, and *CYPs* from *I. amethystoides* were translated into amino acid sequences using SnapGene software. Previously characterized homologous proteins were retrieved from the National Center for Biotechnology Information (NCBI) database (https://www.ncbi.nlm.nih.gov/, Table S3, Table S4). Multiple sequence alignments were performed using ClustalW. Maximum likelihood (ML) phylogenetic trees were constructed in MEGA 11 software [32], employing 1000 bootstrap replicates to assess branch reliability. The resulting phylogenetic trees were visualized and annotated using the Interactive Tree Of Life (iTOL) online tool (https://itol.embl.de/) [33].

### 2.5 Characterization of IamCPSs and IamKSLs in an *Escherichia coli* expression system

A metabolic engineering system in C41 *E. coli* strains overexpressing *dxs*, *dxr*, *idi*, and GGPP synthase from *Abies grandis* (AgGPPS) [34] was employed to enhance production of the universal precursor GGPP [35,36]. Accordingly, the *IamCPS*s and *IamKSL*s were cloned into the pACYCDuet and pET32a vectors (Table S1), respectively. For functional characterization of candidate diTPSs, previously characterized diTPSs were used as positive controls. ZmCPS2/An2 from *Zea mays* produces *ent-*CPP, whereas AtKS from *Arabidopsis thaliana* converts *ent-*CPP to *ent-*kaurene. In addition, the bifunctional diTPS PpCPS/KS and its mutant PpCPS/KS:I741T were used to directly convert GGPP into *ent-*kaurene, 16α-hydroxy-*ent-*kaurane, and *ent-*pimara-8(14),15-diene. The PpCPS/KS:I741T mutant was generated by overlap extension PCR with gene-specific primers, using plasmid pD2 (PpCPS/KS cloned into the pET32a vector) as the template, and the two PCR fragments were then assembled to construct the recombinant plasmid pD3.

The recombinant plasmids carrying distinct selectable markers were co-transformed into C41 strain by electroporation and cultured on resistant plate at 37 °C overnight (100 mg/L ampicillin, 66 mg/L spectinomycin, and 33 mg/L chloramphenicol). Positive transformants were verified by colony PCR using the 2F-DuetUP2/T7-ter and S-Tag/T7-ter primer pairs. Verified positive strains were first cultured in 10 mL of Terrific Broth (TB) medium (12 g/L casein acid hydrolysate, 24 g/L yeast extract, 0.4% v/v glycerol, 17 mM KH_2_PO_4_, 72 mM K_2_HPO_4_) supplemented with the appropriate antibiotics in 50 mL shake flask. Primary seed cultures were grown at 37 °C with shaking at 220 rpm until reaching the mid-exponential growth phase (OD_600_ = 0.6–0.8). Heterologous production of the compounds was induced by adding 1 mM isopropyl β*-*d-1-thiogalactopyranoside (IPTG), together with 40 mM sodium pyruvate and 1 mM MgCl_2_ as metabolic supplements. After induction, the fermentation temperature was reduced to 16 °C and the shaking speed to 160 rpm, and the cultures were incubated for an additional 48 h to promote product accumulation.

### 2.6 Candidate CYPs screening, cloning and plasmid construction

Candidate CYPs were selected according to two criteria: fragments per kilobase of transcript per million mapped reads (FPKM) ≥ 5, and co-expression with predicted upstream genes involved in the *ent-*kaurene diterpenoids biosynthesis. *IamCPS1* and *IamCPS5* were used as bait genes. Lists of co-expressed genes were filtered according to Pearson's correlation coefficient (*r* > 0.6). The expression values shown in the heat map are log_2_-transformed transcript abundances, calculated as Trimmed mean of M (TMM)-normalized counts per million (CPM) in Z-score scale.

The full-length sequences of the candidate genes were amplified from cDNA of *I. amethystoides* with gene-specific primers listed in Table S2 using KOD One™ PCR Master Mix-Blue-Polymerase. The PCR products were purified from agarose gels and ligated into the linearized expression vectors, which were then transformed into *E. coli* DH5α cells. Recombinant colonies were selected on Luria−Bertani (LB) agar plates supplemented with ampicillin (100 mg/L). Plasmids were extracted from positive clones and verified by Sanger sequencing.

### 2.7 Microsome assays and enzymatic assays of candidate CYPs

All candidate *CYP*s were cloned into pESC-URA expression vector with *GAL1* promoter to initiate the transcription. The resulting constructs were individually transformed into strain WAT11 (harboring ATR1) using the Frozen EZ Yeast Transformation II Kit™ (Zymo Research, Irvine, CA, USA). For microsome preparation, a single colony of transformed yeast cells was inoculated into 10 mL SD-URA medium containing 2% glucose and cultured overnight at 30 °C with shaking at 200 rpm. The next day, 2 mL of the overnight culture was used to inoculate 100 mL of fresh SD-URA medium containing 2% glucose, and the cultures were grown until the OD_600_ reached 0.8‒1.0. The yeast cells were then harvested by centrifugation, resuspended in 200 mL of YPL induction medium (1% yeast extract, 2% peptone, and 2% galactose), and incubated at 30 °C with shaking at 160 rpm for 16 h to induce recombinant CYP protein expression.

Cell pellets were collected by centrifugation and resuspended in 10 mL of TEK buffer (50 mM Tris-HCl, pH = 7.5, 1 mM EDTA, and 0.1 M KCl) with thorough vortexing. After incubation at room temperature for 5 min, the cells were pelleted again by centrifugation (4000 *g*, 5 min, 4 °C). The pellets were then resuspended in 30 mL TESB buffer (50 mM Tris-HCl, pH = 7.5, 1 mM EDTA, and 0.6 M sorbitol) and chilled on ice for 10 min. Cell disruption was performed using a nano homogenizer machine (ATS Engineering Limited) at 12,000 psi (7 min, 4 °C). The lysates were clarified by centrifugation (12,000 *g*, 15 min, 4 °C). The supernatants were mixed with an equal volume of ice-cold TESB buffer (0.3 M NaCl and 0.2 g/mL of PEG4000) and incubated on ice for 15 min. Microsomes were collected by centrifugation (12,000 *g*, 15 min, 4 °C), resuspended in TEG buffer (50 mM Tris-HCl, pH = 7.5, 1 mM EDTA, 20% glycerol), and stored at −80 °C.

The reaction mixture consisted of 60 μL of microsomes, 1 mM NADPH, 5 μM FAD, 5 μM FMN, 5 mM glucose-6-phosphate, 1 U/mL glucose-6-phosphate dehydrogenase, 100 μM substrate, and TE buffer (50 mM Tris-HCl, pH 7.5, 1 mM EDTA) in a total volume of 300 μL. Reactions were incubated at 30 °C with shaking at 200 rpm for 2 h. After incubation, the reactions were extracted three times with 300 μL ethyl acetate (EA) each time. The combined organic phase was vaporized to dryness, reconstituted in 100 μL HPLC-grade EA, and analyzed by gas chromatography-mass spectrometry (GC-MS) as described previously.

### 2.8 Construction and engineering of *S. cerevisiae* strains

Strain YC14 was constructed from strain C20 [29] by metabolic engineering to enhance *ent-*kaurene production (Fig. 1D). The bifunctional *JsKS* and the maltose-binding protein (MBP) tag were fused via a flexible “GGGGGGS” peptide linker and the stop codon of *JsKS* were removed. The truncation of gene at N-terminal was predicted using the TMHMM-2.0 online tool (https://services.healthtech.dtu.dk/services/TMHMM-2.0/) [37]. Two truncated *JsKS* variants, designated *t85JsKS* and *t126JsKS*, were generated by deleting the N-terminal transit peptides spanning residues M1–S85 and M1–I126, respectively.

For guide RNA (gRNA) construction targeting the *IME4*p, the N20 sequence of *IME4*p with the second-highest score was selected. Guided by this *IME4*p gRNA, the endogenous promoter of the *IME4* gene was precisely excised via CRISPR-Cas9-mediated cleavage and replaced with heterologous promoters. The 20-nucleotide target sequence-containing gRNA oligos were designed using the CHOPCHOP webtool (http://chopchop.cbu.uib.no) [38] and cloned into the p426 vector. The resulting promoter fragments and corresponding gRNA plasmids were co-transformed into the yeast strain, and transformants were selected on SD-URA dropout plates. All engineered strains were verified by PCR analysis, and the gRNA plasmids were cured by culturing the strains on SC + 5-fluoroorotic acid (5 FOA) plates. In addition, the bifunctional *ent-KS* and *ATR1* genes were integrated into their designated chromosomal loci to enable stable and high-level expression of the heterologous genes.

### 2.9 Fermentation of *ent-*kaurene and 3α,15β-dihydroxy-*ent-*kaurene (3) in shake flask

For fermentation of the genomic-integrated strains, cells were precultured in plastic 96-deep well plates containing 200 μL YPD medium (10 g/L of yeast extract, 20 g/L of peptone, and 20 g/L of glucose) at 30 °C, 500 rpm in a Multitron shaker (www.zhichengyp.com, model ZWY-200D). After incubation for 24 h, the seed cultures were inoculated into 50 mL shake flask with 5 mL of YPD medium at an initial OD_600_ of 0.1 and cultivated at 30 °C, 200 rpm for 72 h before metabolite extraction.

### 2.10 Metabolite extraction and GC-MS analysis

The same extraction and quantification protocols were applied to all engineered strains. Briefly, 1 mL of culture was extracted three times with 1 mL of EA by vortexing, followed by centrifugation at 12,000 g for 5 min. All supernatant was transferred to a clean vial and evaporated to complete dryness, then redissolved in 100 μL of HPLC-grade EA for GC-MS analysis. GC-MS analysis was performed using Thermo TRACE 1310/TSQ 8000 gas chromatograph equipped with a TG-5 MS (30 m × 0.25 mm × 0.25 μm) capillary column. Samples were injected in split mode (25:1) at 280 °C under a helium flow rate of 1 mL/min. The GC oven temperature gradient was as follows: 50 °C, holding for 1 min; 50 °C–220 °C, 50 °C/min; 220 °C–270 °C, 10 °C/min; 270 °C–300 °C, 40 °C/min; 300 °C, holding for 3 min. The ion trap heating temperature was 280 °C. The electron energy was 70 eV. The scan range was 50–500 *m/z*.

## 3 Results

### 3.1 Metabolites quantification and transcriptome analysis of *I. amethystoides*

**Fig. 2:**
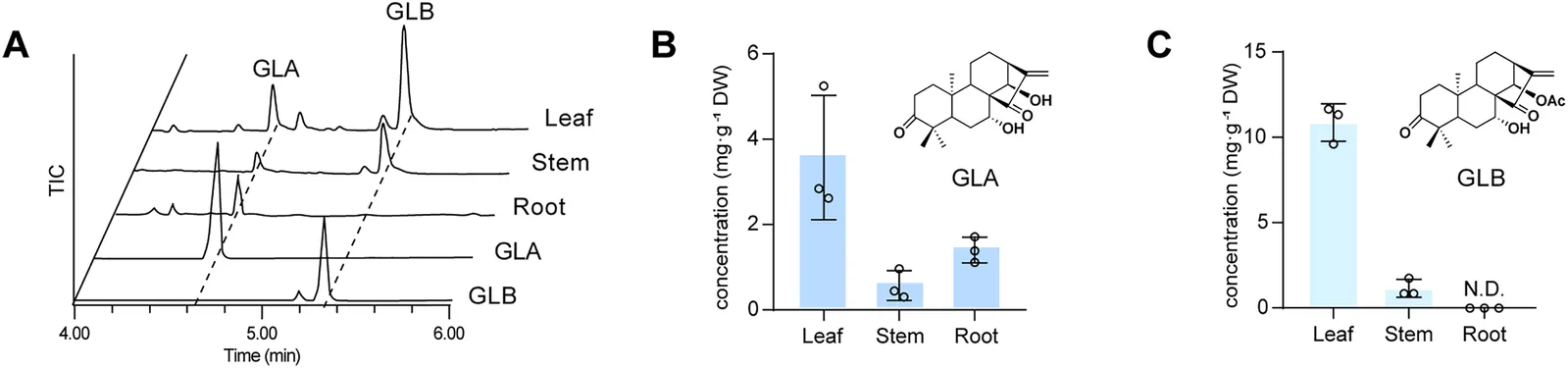
Accumulation pattern of GLA and GLB in *I. amethystoides*. (A) UPLC analysis of GLA and GLB extracted from *I. amethystoides*. (B) The concentration of GLA in *I. amethystoides*. (C) The concentration of GLB in *I. amethystoides*. Note that GLB was not detected (N.D.) in roots. Data are presented as mean values ± SD (n = 3 biologically independent replicates).

Given the conserved spatiotemporal patterns of metabolite accumulation and biosynthetic gene expression across plant tissues [39], we adopted a metabolite-guided transcriptomic approach to identify genes involved in the GLA biosynthetic pathway. First, the contents of GLA and GLB were quantified by UPLC analysis (Fig. 2A). GLA primarily accumulated in leaves (3.6 ± 1.5 mg/g dry weight), while its abundance was markedly lower in stems and roots (Fig. 2B). GLB showed the highest accumulation in leaves (10.9 ± 1.1 mg/g dry weight), which was 9.5-fold higher than that in stems (1.14 ± 0.5 mg/g dry weight), and it was not detected in roots (Fig. 2C).

Total RNA extracted from the leaves, stems, and roots of *I. amethystoides* was used for RNA-Seq analysis (Fig. S2A). The RNA-seq database was assembled into 62,745 unigenes with an average contig length of 1803 bp. Of these unigenes, 75.29% were annotated in at least one database (NR, NT, KO, Swiss-Prot, PFAM, GO, KOG), while 10.85% were annotated in all databases (Fig. S2B). KEGG annotation of 22,025 genes revealed their distribution across five primary metabolic pathways, with 347 genes (1.58%) involved in terpenoid and polyketide metabolism (Fig. S2C).

### 3.2 Identification of IamCPS1 and IamCPS5 involved in *ent-*kaurene biosynthesis of *I. amethystoides*

**Fig. 3:**
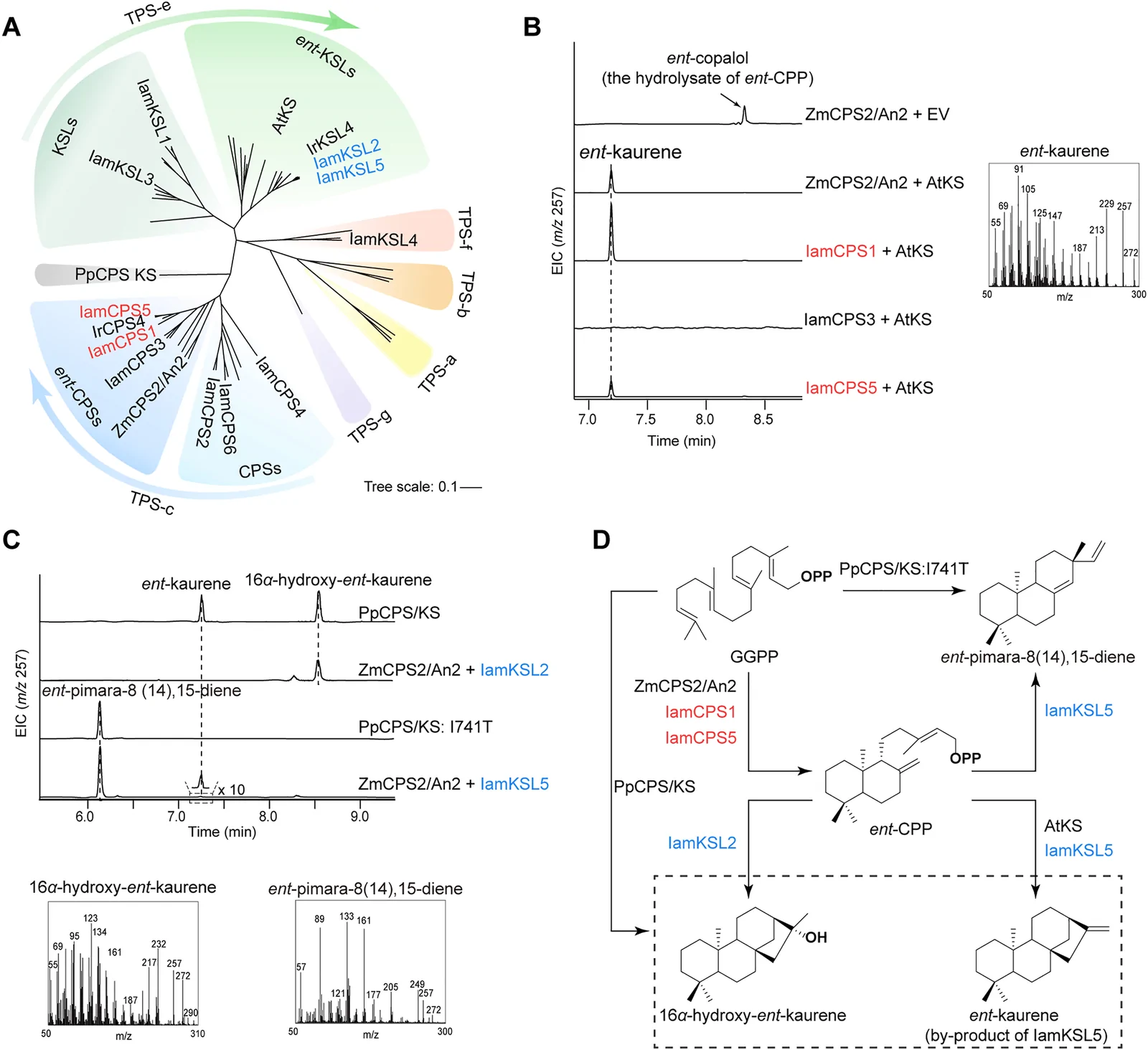
Identification of *ent*-CPSs and *en*t-KSLs for *ent*-kaurene diterpenoids biosynthesis in *I. amethystoides*. (A) Phylogenetic analysis and characterization of diterpenoid synthases. (B) Characterization of IamCPS1, IamCPS3 and IamCPS5. ZmCPS2/An2 was used as positive control for *ent-*CPP production. AtKS was served as positive control which catalyzes the conversion of *ent-*CPP to *ent-*kaurene. (C) Characterization of IamKSL2 and IamKSL5. PpCPS/KS can directly convert GGPP into *ent-*kaurene, while its mutant PpCPS/KS:I741T can directly convert GGPP into 16α*-*hydroxy-*ent-*kaurane and *ent-*pimara-8(14),15-diene. All extracted metabolites were shown in EIC mode (*m/z* 257). (D) Schematic diagram of the *ent*-CPSs and *ent*-KSLs involved in the formation of *ent-*kaurene in *I. amethystoides*.

The biosynthesis of *ent-*kaurene requires *ent-*CPSs and *ent-*KSLs, or alternatively bifunctional diTPSs [40]. Transcriptomic analysis identified 11 full-length diTPSs, including 6 CPSs and 5 KSLs (Fig. S3). Phylogenetic analysis revealed that IamCPS1, IamCPS3 and IamCPS5 clustered with the functionally characterized IrCPS4 and IrCPS5 (Fig. 3A, Fig. S4) [41]. Meanwhile, IamKSL2 and IamKSL5 grouped with IrKSL4, an enzyme known to utilize *ent-*CPP as a substrate to produce *ent-*atiserene and *ent-*kaurene [41], indicating their pivotal roles in the *ent-*kaurene biosynthetic pathway (Fig. 3A, Fig. S4).

IamCPS1, IamCPS3 and IamCPS5 were functionally characterized via *in vitro* assays in combination with the *ent-*kaurene synthase AtKS. GC-MS analysis revealed that the IamCPS1-AtKS and IamCPS5-AtKS combinations catalyzed the conversion of GGPP into *ent-*kaurene, while no activity was detected for IamCPS3 in combinatorial assays with AtKS. These results confirmed that both IamCPS1 and IamCPS5 function as *ent*-CPP synthases, with the IamCPS5-AtKS combination producing a higher yield of *ent*-kaurene (Fig. 3B and D). In addition, IamKSL2 catalyzed the conversion of *ent-*CPP into 16α*-*hydroxy-*ent-*kaurene, while IamKSL5 predominately produced *ent-*pimara-8 (14),15-diene, with *ent*-kaurene as a minor by-product (Fig. 3C and D), consistent with the activities of the functionally characterized enzymes PpCPS/KS and PpCPS/KS:I741T [42,43].

### 3.3 Co-expression analysis of candidate CYPs with IamCPS1 and IamCPS5

**Fig. 4:**
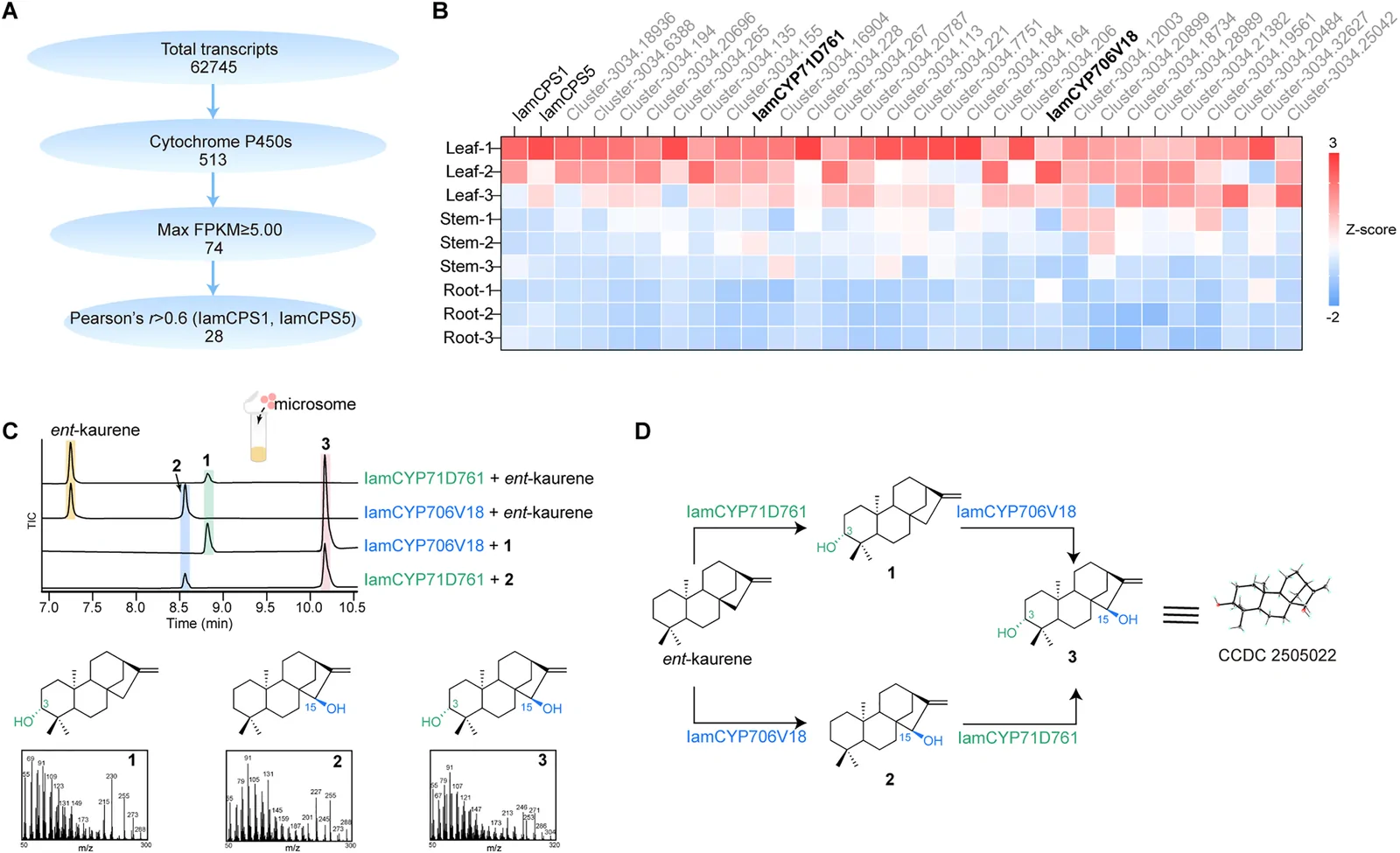
IamCYP71D761 and IamCYP706V18 are responsible for the C3α- and C15β-hydroxylation of *ent*-kaurene in *I. amethystoides*. (A) Sequential filtering of candidate *CYP* genes from the *I. amethystoides*. (B) The heatmap displays expression levels (Z-score scaled) across nine samples from three tissues (leaf, stem, root). (C) Microsome assays of IamCYP71D761 and IamCYP706V18. GC-MS profiles and MS data were shown. (D) Schematic diagram of the catalytic reactions.

Recently, co-expression analysis combined with comparative transcriptomics has been developed to mine the enzymes involved in plant biosynthetic pathways. From a total of 62,745 transcripts, 513 annotated CYPs were retrieved. Of these, 74 CYPs with an FPKM value ≥ 5 were retained for further analysis. To prioritize candidate CYPs potentially responsible for *ent-*kaurene hydroxylation, we performed co-expression analysis using *IamCPS1* and *IamCPS5* as bait genes, with a Pearson correlation coefficient threshold of *r* > 0.6. *IamCPS1* retrieved a cluster of 25 CYPs, and *IamCPS5* retrieved another cluster of 25 CYPs. Among these, 22 CYPs were common to both clusters, whereas each bait gene yielded three unique CYPs that did not overlap with another cluster. Thus, a combined set of 28 CYPs was predicted to be involved in the hydroxylation of the *ent-*kaurene skeleton (Fig. 4A, Fig. S5). Notably, all candidate CYP genes exhibited higher transcript levels in leaves, which is consistent with the accumulation patterns of compounds GLA and GLB across different tissues (Fig. 4B).

### 3.4 IamCYP71D761 and IamCYP706V18 are responsible for the C3α- and C15β-hydroxylation of ent-kaurene in *I. amethystoides*

We cloned the 28 CYP candidates from cDNA using specific primers, inserted them into pESC-URA plasmid, and expressed in the *S. cerevisiae* WAT11 strain (harbors ATR1) [44], generating recombinant strains for *in vitro* experiments. Microsomes were prepared and incubated with *ent-*kaurene followed by GC-MS analysis. IamCYP71D761 converted *ent-*kaurene into a new product **1** (*m/z* 288), showing an increase of 16 Da compared to *ent-*kaurene (*m/z* 272). IamCYP706V18 yielded compound **2**, and mass spectrometry (MS) analysis showed an *m/z* of 288, indicating the introduction of a hydroxyl group (Fig. 4C). The structures of compounds **1** and **2** were identified by NMR spectroscopy as 3α-hydroxy-*ent-*kaurene and 15β-hydroxy-*ent-*kaurene, respectively (Fig. S12 and 13).

Double-hydroxylated *ent-*kaurene was detected when IamCYP706V18 was incubated with compound **1** or when IamCYP71D761 was incubated with compound **2** (Fig. 4C). The structure and stereochemistry of product **3** was unequivocally confirmed by NMR spectroscopy (Fig. S14) and X-ray crystallography (CCDC 2505022; Fig. 4D). These *in vitro* enzymatic results showed that IamCYP71D761 and IamCYP706V18 could alternately oxidize the *ent-*kaurene skeleton to produce **3** via two branching pathways (Fig. 4D). Kinetic analysis demonstrated that IamCYP71D761 exhibited both higher substrate affinity and greater catalytic efficiency for converting **2** into **3** than for converting *ent-*kaurene into **1** (Fig. S6A and B). IamCYP706V18 showed higher substrate affinity for *ent-*kaurene, while exhibiting greater catalytic efficiency towards compound **1** (Fig. S6C and D).

To further elucidate the catalytic properties of IamCYP71D761 and IamCYP706V18, the ketone products 3-carbonyl-*ent-*kaurene (**4**) and 15-carbonyl-*ent-*kaurene (**5**) were synthesized by pyridinium chlorochromate (PCC) oxidation of the corresponding hydroxylated compounds (Fig. S7A and B). Products **4** and **5** were purified and identified by NMR spectroscopic analysis (Fig. S15 and 16). IamCYP706V18 showed C15β*-*hydroxyl regioselectivity toward **4**, yielding 15-carbonyl-3α*-*hydroxy-*ent-*kaurene (Fig. S7C), while IamCYP71D761 selectively hydroxylated the C3 position of **5** to produce 3-carbonyl-15β*-*hydroxy-*ent-*kaurene (Fig. S7D). Collectively, these results demonstrate that IamCYP71D761 and IamCYP706V18 exhibit substrate cross-recognition and catalyze convergent oxidation pathways, extending to downstream steps via the key precursor **3** (Fig. S7E).

### 3.5 Heterologous reconstitution of *ent-*kaurene biosynthetic pathway in *S*. *cerevisiae*

To establish a robust yeast cell factory for efficient production of the key precursor *ent*-kaurene, we reconstituted its heterologous biosynthetic pathway in *S. cerevisiae* (Table 1). The previously engineered GGPP-producing strain C20, which carries an enhanced MVA pathway, was adopted as the parent chassis for *ent*-kaurene biosynthesis [29]. A GGPP synthase (XdGGPPS) from *Xanthophyllomyces dendrorhous* was further integrated into the *XII-5* locus of C20 genome, yielding the engineered strain YC1 as the basal platform for subsequent metabolic engineering.

**Fig. 5:**
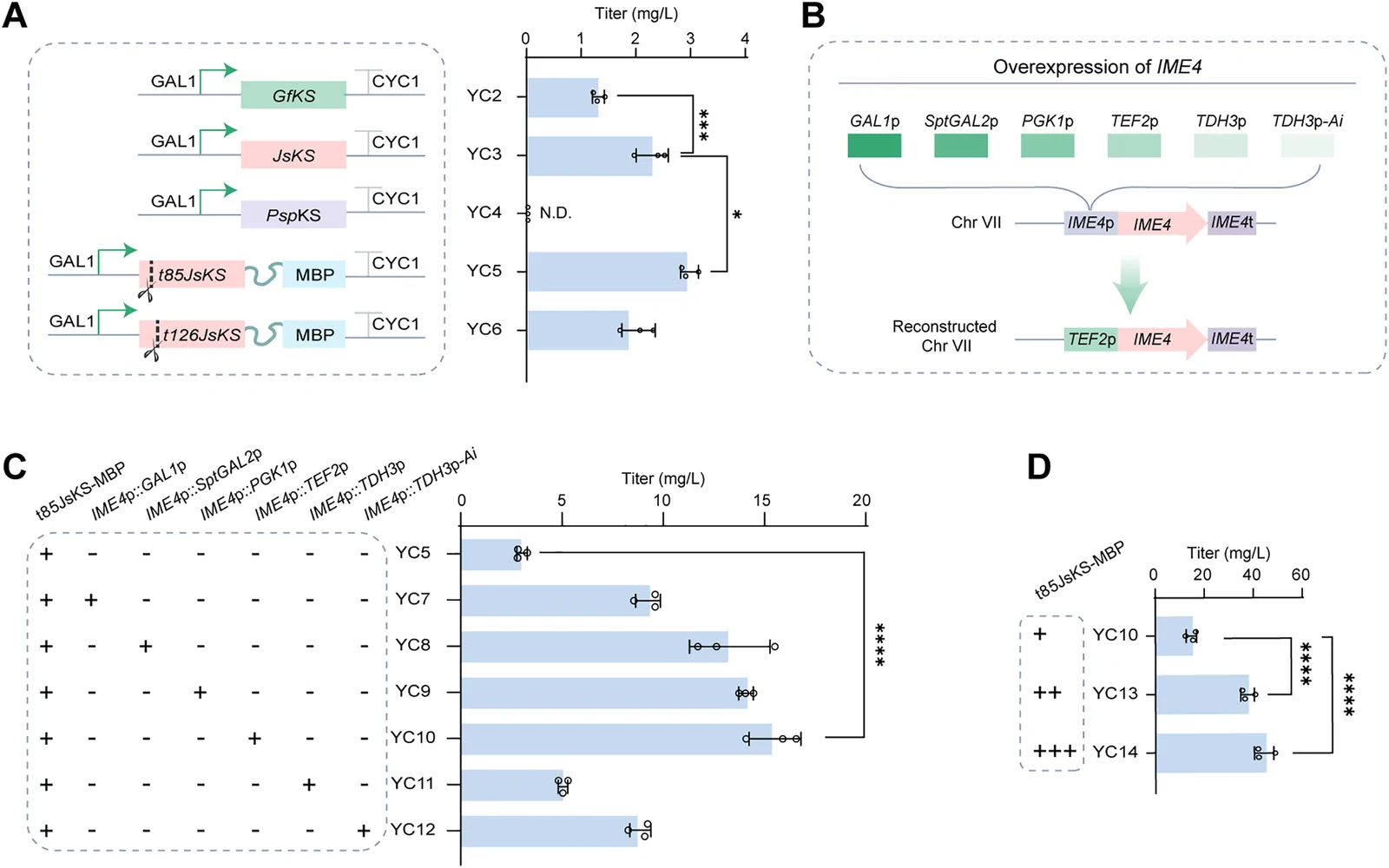
Heterologous reconstitution of *ent-*kaurene biosynthetic pathway in *S. cerevisiae*. (A) Screening the bifunctional *ent-*KS with better performance on *ent-*kaurene synthesis in chassis strain YC01. (B) Schematic diagram of promoter engineering design for modulating endogenous IME4 expression to increase acetyl-CoA supply. (C) Engineered strains that overexpress *IME4* for *ent-*kaurene production. (D) Engineered strains harboring different copy numbers of *t85JSKS-MBP* for *ent-*kaurene production. Data are presented as mean values ± SD (n = 3 biologically independent replicates). Statistical significance of each strain is shown (****P < 0.0001, ***P < 0.001, *P < 0.05).

In strain YC1, we reconstructed the *ent*-kaurene biosynthetic route directly from GGPP by introducing bifunctional diterpene synthases. Three previously reported diterpene synthases, namely GfKS from *Gibberella fujikuroi* [45], JsKS from *Jungermannia subulate* [46], and PspKS from *Phaeosphaeria sp.* L487 [47] were individually introduced into YC1 to yield strains YC2, YC3, and YC4. Strain YC3 carrying JsKS was selected for further optimization due to its higher *ent-*kaurene titer (2.3 ± 0.3 mg/L) (Fig. 5A). Inspired by previous reports [48,49], we adopted a dual engineering strategy: C-terminal MBP tagging to improve protein stability and solubility, and N-terminal truncation to eliminate predicted signal peptides. The resulting truncated variants t85JsKS-MBP and t126JsKS-MBP were expressed to generate strains YC5 and YC6. Among them, YC5 expressing t85JsKS-MBP demonstrated superior performance, with the *ent*-kaurene titer reaching 3.0 ± 0.4 mg/L (Fig. 5A).

**Fig. 6:**
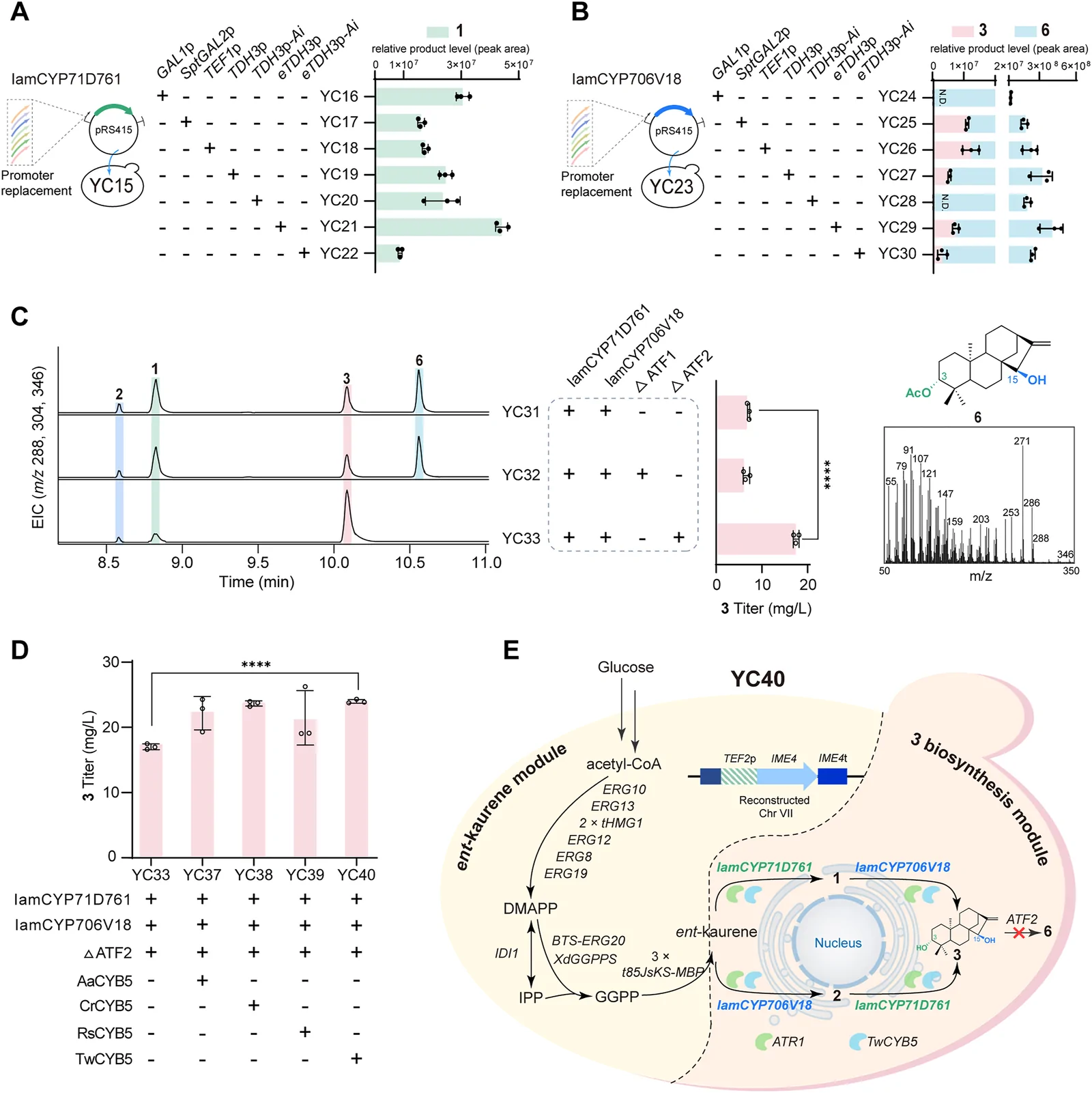
Construction and optimization of the *de novo* biosynthetic pathway for **3** in *S. cerevisiae*. (A) Relative product level of **1** catalyzed by IamCYP71D761 under different promoter regulations. (B) Relative product levels of **3** and **6** catalyzed by IamCYP706V18 under different promoter regulations. (C) Knockout of endogenous acetyltransferase *ScATF1* or *ScATF2* to inhibit the accumulation of acetylation by-products. Strain YC33 exhibited an improved titer of **3**. (D) Production of **3** in engineered strains. Data are presented as mean values ± SD (*n* = 3 biologically independent replicates). Statistical significance of each strain is shown (****P < 0.0001). (E) Schematic diagram of the engineered biosynthetic pathway for **3** in strain YC40.

Sufficient precursor supply is essential for efficient downstream biosynthetic modification. The yeast m^6^A methyltransferase (IME4) serves as the catalytic member for the methylation to the N^6^ position of adenosine residues in messenger RNA (mRNA), generating N^6^-methyladenosine (m^6^A) modifications. This modification is crucial for post-transcriptional regulation of gene expression. The IME4 overexpression has been reported to upregulate the expression levels of key genes involved in acetyl-CoA biosynthesis [50]. Thus, we adopted a promoter engineering strategy to modulate IME4 expression. The native promoter of IME4 was individually replaced with two inducible promoters (*GAL1*p and *SptGAL2*p [51] from *S. pastorianus*), three constitutive promoters (*PGK1*p, *TEF2*p, and *TDH3*p), and one chimeric constitutive promoter *TDH3*p*-RPS25Ai* (*TDH3*p*-Ai*). This chimeric promoter was constructed by fusing *TDH3*p with the intron region of the *RPS25A* promoter [52], generating a series of engineered strains YC7–YC12 (Fig. 5B and C). Strain YC10 achieved a 5.2-fold increase in *ent*-kaurene production compared to the parent strain YC5, with a titer of 15.4 ± 1.7 mg/L. To further enhance *ent-*kaurene production, two additional copies of t85JsKS-MBP were overexpressed to consume the accumulated GGPP precursor. This modification further elevated the *ent*-kaurene titer to 45.3 ± 3.0 mg/L in strain YC14 (Fig. 5D). Collectively, the optimized *ent*-kaurene biosynthetic module was successfully assembled, facilitating the *de novo* biosynthesis of **3** in yeast (Table S2, Fig. 6D).

### *3.6* *De Novo* biosynthesis of 3α,15β*-*dihydroxy-*ent-*kaurene (3) in *S. cerevisiae*

To construct the biosynthetic module for compound **3**, the *ATR1* gene was introduced into strain YC14 to provide electron transfer support, yielding strain YC15 (Table 1). To investigate the CYP proteins expression *in vivo*, fusion expression vectors of the *IamCYP71D761* and *IamCYP706V18* genes with *GFP* were constructed and expressed in CEN.PK2-1D. Specific bands at the expected molecular weights were observed using western blot analysis with GFP antibodies (Fig. S8). Promoter engineering is widely applied across eukaryotes including yeast, plants and mammals, and serves as a pivotal strategy for modulating gene expression [[51], [52], [53]]. Previous studies have demonstrated that intron-containing promoters generally drive stronger gene expression than the conventional *TDH3* promoter (*TDH3*p) [52]. Here, we selected a promoter library covering a broad expression dynamic range, including *SptGAL2*p, *GAL1*p, *TEF1*p, *TDH3*p alongside three additional synthetic variants *eTDH3*p, *TDH3*p*-RPS25Ai* (*TDH3*p-*Ai*) and *eTDH3*p*-RPS25Ai* (e*TDH3*p-*Ai*) [54]. Plasmids pL1–pL7, each carrying *IamCYP71D761* driven by one of the seven promoters (Table S1), were individually transformed into YC15 to generate strains YC16–YC22. Strain YC21 harboring the *eTDH3*p variant achieved the highest product titer (Fig. 6A). Thus, *IamCYP71D761* controlled by *eTDH3*p was integrated into the genomic *1414a* locus of YC15 to construct strain YC23.

Subsequently, plasmids pL8–pL14 carrying *IamCYP706V18* under the control of the same seven promoters (Table S1) were transformed into YC23, producing strains YC24–YC30. Among these, *eTDH3*p-driven expression in strain YC29 afforded the optimal catalytic conversion efficiency (Fig. 6B). Following the same strategy, *IamCYP706V18* with *eTDH3*p was integrated into the genomic *106a* loci of YC23 to generate strain YC31.

Co-expression of the two optimized CYP expression cassettes in YC31 enabled the *de novo* biosynthesis of compound **3** at a titer of 6.7 ± 0.1 mg/L. Meanwhile, GC-MS detection identified compound **1**, compound **2**, and an additional novel product **6** (Fig. 6C). Compound **6** (*m/z* 346) exhibited a molecular ion at *m/z* 346, showing a 42 Da mass increase compared to **3**, which matched the molecular mass of an acetyl group (Fig. 6C). To clarify its chemical structure, we performed scale-up fermentation, isolation and purification, Compound **6** was confirmed as 3-acetoxyl-15β*-*hydroxyl-*ent-*kaurene based on ^1^H NMR and ^13^C NMR analysis (Fig. S17).

To redirect metabolic flux toward the accumulation of compound **3**, we disrupted endogenous acetyltransferase genes previously reported to mediate terpenoid acetylation [55,56]. We hypothesized that knockout of these acetyltransferases would attenuate the conversion of **3** to its acetylated derivative **6**. Two alcohol *O*-acetyltransferase genes, *ScATF1* and *ScATF2*, were individually deleted to generate strains YC32 and YC33, respectively. As expected, deletion of *ScATF2* completely eliminated the accumulation of acetylated product **6** and increased the titer of **3** to 17.4 ± 0.3 mg/L in strain YC33, representing a 2.6-fold improvement compared with YC31, and *in vitro* enzymatic assays confirmed that ScATF2 catalyzes the conversion of **3** to **6** (Fig. S9). In contrast, *ScATF1* knockout showed no significant impact on product profiles (Fig. 6C).

To further increase the titer of **3**, we generated strains YC34–YC36 with increased copy numbers of IamCYP71D761 or IamCYP706V18. Rather than boosting the yield of compound **3**, the presence of extra CYP copies led to the buildup of monohydroxylated intermediates **1** and **2** (Fig. S10). Such results suggest that the protein expression levels of these two CYPs are already sufficient. We therefore shifted our attention to the electron transfer system, which is critical for P450 catalytic function. Cytochrome *b*5 (CYB5) serves as a key electron donor partner for plant P450s, and we reasoned that enhancing electron supply might improve the overall conversion efficiency. Accordingly, we introduced cytochrome *b*5 (CYB5) from four different species (*Artemisia annua*, *Catharanthus roseus*, *Rubus suavissimus*, and *Tripterygium wilfordii*) [29] into strain YC33 to generate strains YC37–YC40 (Fig. 5D). Overexpression of TwCYB5 in strain YC40 achieved the highest titer of **3**, reaching 24.0 ± 0.2 mg/L in shake-flask cultivation (Fig. 5D and E).

## 4 Discussion and conclusions

CYPs have evolved to catalyze diverse structural post-modifications of diterpenoids. In the present study, two CYP enzymes with C3α- and C15β-hydroxylation toward *ent*-kaurene were functionally characterized. Phylogenetic analysis revealed that IamCYP71D761 and IamCYP706V18 belong to the CYP71 clan and cluster with previously characterized diterpenoid-modifying CYPs (Fig. S11) [19,23,26]. Biochemical assays demonstrated that IamCYP71D761 efficiently catalyzes C3α-hydroxylation of *ent*-kaurene, as well as its C15-hydroxylated and C15-aldehydated derivatives. The findings broaden the substrate spectrum of the CYP71D clan and further demonstrate its functional plasticity in *ent*-kaurene diterpenoid modification. Furthermore, previous studies have demonstrated that tandem-duplicated CYP706V oxidase from *I. rubescens* participate in oridonin biosynthesis by mediating C7β, C14β, and C15β hydroxylation. Although the CYP706V subfamily possesses great potential for *ent*-kaurene diterpenoid modification, its natural distribution is currently restricted to *Lamiaceae* species, including *I. rubescens* and *Sesamum Indicum* [26]. In this work, IamCYP706V18 was proven to recognize both *ent*-kaurene and its C3-modified derivatives. The functional identification of IamCYP706V18 as a specific C15β-hydroxylase not only enriches the functional diversity of the CYP706V subfamily, but also highlights *Isodon* species as a valuable genetic reservoir for oxidative modification enzymes.

The capability of IamCYP71D761 and IamCYP706V18 to recognize both *ent*-kaurene scaffold and its oxidized intermediates reflect the conserved “metabolic grid” commonly present in plant specialized metabolism [29,49,57]. The cross-recognition pattern may represent a prevalent metabolic strategy to expand structure diversity with limited enzymatic resources. Despite the progress in identifying key hydroxylation enzymes, which are responsible for the C7α- and C14β-hydroxylation steps in GLA biosynthesis remain uncharacterized. Considering the previously reported low catalytic efficiency of CYP706V members toward *ent*-kaurene [26], we speculate that these two oxidation reactions occur at the late biosynthetic stage. Correspondingly, these modifications are more likely to target partially oxidized intermediates, such as the dihydroxylated product **3** and ketonized product, rather than the unmodified *ent*-kaurene skeleton. Although compound **3** was successfully produced in heterologous systems, it was not detected in *I. amethystoides*. We attribute this absence to its rapid metabolic turnover by downstream enzymes in GLA/GLB biosynthesis pathway, which is consistent with its proposed role as a transient intermediate rather than an accumulating metabolite.

In this work, through systematical engineering strategies, including rational protein design (MBP fusion and N-terminal truncation), upregulation of the TCA cycle via IME4 overexpression, promoter-driven fine-tuning of CYP expression, knockout of the endogenous *ATF2* gene, and introduction of heterologous TwCYB5 to enhance electron transfer, we developed strain YC40, which stably produces **3** at 24.0 ± 0.2 mg/L in shake flask. To our knowledge, this is the first report of *de novo* biosynthesis of **3** from glucose in *S. cerevisiae*. In addition, the established platform enables the scalable and sustainable production of *ent*-kaurene intermediates, paving the way for the *de novo* synthesis of GLA, GLB, and other high-value diterpenoids. Although the engineered yeast strain achieved *de novo* production of **3**, the final titer of 24.0 ± 0.2 mg/L remains relatively low for industrial applications. Further improvement of the product titer remains challenging. The primary bottleneck lies in insufficient intracellular supply of the *ent*-kaurene precursor. Our preliminary metabolic engineering approaches, including modulating endogenous IME4 expression to increase acetyl-CoA supply [50] and elevating the copy number of t85JsKS-MBP, substantially increased *ent*-kaurene production from 3.0 ± 0.4 mg/L to 45.3 ± 3.0 mg/L (Fig. 5C and D), These outcomes imply that further optimization of acetyl-CoA biosynthesis and increasing the copy number of t85JsKS-MBP may likely result in a continued improvement in yield [58]. Ma et al. introduced a two-step isopentenol utilization pathway (IUP) into *S. cerevisiae* to augment the native MVA pathway, which elevated the titers of industrially important terpenoids by several folds [59]. Inspired by this report, we propose that incorporation of the IUP pathway into our engineered strain can effectively augment intracellular GGPP precursor availability. Another bottleneck lies in the catalytic microenvironment of the P450 system. Large amounts of unconsumed monohydroxylated intermediates, particularly the C3α-hydroxylated **1**, were still detectable in strain YC40 (Fig. S10). Notably, increasing the copy number of the two P450 genes failed to boost the titer of **3**, while introduction of TwCYB5 to reinforce electron delivery drastically elevated product yield. Accordingly, follow-up efforts should prioritize systematic optimization of the P450 catalytic microenvironment by augmenting the intracellular pool of essential cofactors, including electron transport proteins and heme, alongside expanding endoplasmic reticulum storage capacity [60]. In addition, the accumulation level of the C15β-hydroxylated intermediate **1** was consistently lower than that of the C3α-hydroxylated product **2** in YC40 strain, indicating that IamCYP706V18 exhibits insufficient catalytic activity toward the substrate **1**. Therefore, protein engineering of IamCYP706V18 represents a highly viable strategy for further improving the yield.

## CRediT authorship contribution statement

**Yanying Chen:** Writing – review & editing, Writing – original draft, Validation, Methodology, Investigation, Formal analysis, Data curation. **Xinqi Song:** Validation, Software, Methodology, Investigation, Formal analysis, Data curation. **Yanan Wang:** Formal analysis, Data curation. **Yang Han:** Methodology, Investigation, Formal analysis. **Meng Xia:** Methodology, Formal analysis. **Shijun Yuan:** Methodology, Formal analysis. **Ying Ma:** Validation, Methodology. **Jian Wang:** Methodology, Formal analysis. **Qing Ma:** Validation, Methodology. **Shuang Liu:** Writing – review & editing, Resources, Project administration, Funding acquisition. **Ping Su:** Writing – review & editing, Supervision, Resources, Project administration, Methodology, Funding acquisition. **Luqi Huang:** Supervision, Project administration.

## Declaration of competing interests

The authors declare that they have no known competing financial interests or personal relationships that could have appeared to influence the work reported in this paper.

## Appendix A Supplementary data

The sequences identified in this paper are available from GenBank with the following entry numbers: IamCPS1 (PX896399), IamCPS5 (PX896401), IamKSL2 (PX896403), IamKSL5 (PX896404), IamCYP71D761 (PX896405) and IamCYP706V18 (PX896406). The Supporting Information is available free of charge. Multimedia component 1
