# Supplementary material for: Characterization of two key cytochrome P450 enzymes in Isodon amethystoides and de novo production of 3α,15β-dihydroxy-ent-kaurene in yeast
Source: Synth Syst Biotechnol. 2026 Aug 27;18:63–74. doi: 10.1016/j.synbio.2026.08.004 (PMC13545690; doi:10.1016/j.synbio.2026.08.004)
Supplement: Supplementary file 1 [file mmc1.docx]

Supplementary information

Characterization of two key cytochrome P450 enzymes in *Isodon amethystoides* and *de novo* production of 3α,15β-dihydroxy-*ent*-kaurene in yeast

Yanying Chen^a,b,1^, Xinqi Song^b,1^, Yanan Wang^b^, Yang Han^b^, Meng Xia^b^, Shijun Yuan^b^, Ying Ma^b^, Jian Wang^b^, Qing Ma^b^, Shuang Liu^c,*^, Ping Su^b,*^, Luqi Huang^a,b,*^

^a^ School of Pharmacy, Chengdu University of Traditional Chinese Medicine, Chengdu, 611137, China

^b^ State Key Laboratory for Quality Ensurance and Sustainable Use of Dao-di Herbs, National Resource Center for Chinese Materia Medica, China Academy of Chinese Medical Sciences, Beijing, 100700, China

^c^ Experimental Research Center, China Academy of Chinese Medical Sciences, Beijing, 100700, China

*Correspondence: Shuang Liu ([scarlett0706@163.com](mailto:scarlett0706@163.com)); Ping Su ([suping120@nrc.ac.cn](mailto:suping120@nrc.ac.cn)); Luqi Huang ([huangluqi01@126.com](mailto:huangluqi01@126.com))

**Supplementary Methods**

**Isolation and purification of compounds 1‒6.**

A 10 mL aliquot of overnight culture of strain YC31 producing compounds **1**, **2**, **3**, and **6** in YPD medium was used to inoculate 1 L YPD medium and grown at 30 °C, 200 rpm for 5 days. The culture was extracted three times with 1 L ethyl acetate (EA). Combined organic layers were harvested by centrifugation and concentrated to yield oily crude. The crude was mixed with twice its weight of 60–100 mesh silica gel and loaded onto a column packed with an 8-fold weight of 100–200 mesh silica gel. The column was eluted with a stepwise gradient of EA/petroleum ether (PE) at ratios of 1:64, 1:48, 1:32, 1:24, 1:16, 1:8, and 1:4 (v/v). The eluates from each gradient step were analyzed by gas chromatography-mass spectrometry (GC-MS) to locate the target compound. Fractions containing the desired products were pooled and further purified by preparative HPLC using a semi-preparation column (SEPARATION Basic C18 column, 120A, 5 μm, 10 mm×250 mm). The samples were eluted with a gradient of a two-solvent mobile phase consisting of water and acetonitrile. The elution was monitored at 220 nm using a diode array detector. Eluates containing the target molecule were then pooled and concentrated. The structures were determined by analyzing their ^1^H and ^13^C NMR spectra. For isolation and purification of compounds **4** and **5**, to a mixture of hydroxylated compound **1** or **2** (7.5 mg, 1.0 equiv.) and pyridinium chlorochromate (PCC) (8.44 mg, 1.5 equiv.), anhydrous dichloromethane (3 mL) was added. The mixture was stirred at 25 °C for 4 hours. The supernatants were collected, dried, and redissolved for separation using preparative HPLC as described above. NMR data described in this study are presented in Fig. S12–S17.

**Enzyme kinetics assays.**

The concentration of active P450 enzyme was determined spectrophotometrically from the reduced CO‑difference spectrum, using an extinction coefficient of 91 mM⁻¹·cm⁻¹ for the absorbance difference between 450 and 490 nm. Kinetic parameters were assessed by incubating the enzyme with varying substrate concentrations ranging from 50 to 2,000 μM in a total reaction volume of 300 μL. The reaction mixture was prepared as described in Section 2.7. The reactions were initiated by the addition of substrate and incubated with shaking at 30°C for 1 h in the dark. Extraction was then terminated three times with 300 μL of EA, and the extracts were completely dried with a vacuum concentrator (Eppendorf Concentrator plus, Germany). The product was quantified using 80 μL of methanol dissolved for GC-MS analysis. The reactions were performed in triplicate (n = 3). Kinetic parameters (*K_m_* and *k_cat_*) were calculated by using GraphPad Prism v9.0.

***In vitro* assays for ScATF2 activity.**

The endogenous gene *ScATF2* was amplified with the specific primers using KOD One^TM^ PCR Master Mix -Blue- (TOYOBO CO., LTD. Life Science Department OSAKA JAPAN) from the lysate of CEN.PK2-1D strain, and subcloned into pET28a vector to generate the pET28a::ScATF2 plasmid. The recombinant plasmid was transformed into *E. coli* BL21 (DE3) cell for expression. The pET28a empty vector was used as the negative control. The transformants were cultured in 50 mL Terrific Broth (TB) medium (12 g/L casein acid hydrolysate, 24 g/L yeast extract, 0.4% v/v glycerol, 17 mM KH_2_PO_4_, 72 mM K_2_HPO_4_) with kanamycin (50 mg/L) at 37 °C until OD_600_ at 0.8‒1.0, then induced with 1 mM isopropyl-β-D-thiogalactopyranoside (IPTG) and grown at 20 °C for 16 h at 150 rpm. The cell pellets were harvested by centrifugation (5,000 g, 10 min, 4 °C), then resuspended in lysis buffer that contained 20% v/v glycerol, 100 mM 4-(2-hydroxyethyl) piperazine-1-ethanesulfonic acid (HEPES), 200 mM KCl, 300 mM NaCl, 20 mM MgCl_2_, 8 mM DTT and sonicated in an ice-water bath for 8 min (lysed for 2 s, paused for 3 s). The sample lysates were centrifuged for 10 min at 10,000 g and 4 °C to separate crude enzymes from cell debris.

The reaction mixture consisted of 490 μL of crude enzymes, 50 μM acetyl-CoA, and 300 μM 3α,15β-dihydroxy-*ent*-kaurene (**3**) in a total volume of 500 μL. Reactions were incubated at 30 °C with shaking at 200 rpm for 12 h. After incubation, the reactions were extracted three times with 500 μL EA each time. The combined organic phase was vaporized to dryness, reconstituted in 100 μL HPLC-grade EA, and analyzed by GC-MS as described previously.

**Western blotting assays.**

The coding sequences of *IamCYP71D761* and *IamCYP706V18* were cloned into pESC-URA vector under the control of the GAL1 promoter to generate fusion expression vectors containing the *GFP* gene. The primers used for vector construction are listed in Table S2. CEN.PK2-1D strains expressing IamCYP71D761-GFP and IamCYP706V18-GFP were cultured in 20 mL SD-URA medium containing 2% glucose and cultured overnight at 30 °C with shaking at 200 rpm. The yeast cells were then harvested by centrifugation, resuspended in 20 mL of YPL induction medium (1% yeast extract, 2% peptone, and 2% galactose), and incubated at 30 °C with shaking at 200 rpm for 16 h to induce recombinant CYP protein expression. Strains were ground with glass beads in liquid nitrogen and centrifuged (12,000 g, 10 min, 4 ℃) to collect the supernatant. The supernatant was denatured in SDS-PAGE loading buffer at 99°C for 5 min. Equal amounts of total protein per sample were separated on SDS-PAGE gels, and proteins were transferred onto a polyvinylidene fluoride membrane (Millipore). The membrane was blocked with 5% nonfat milk to prevent nonspecific binding. A rabbit anti GFP tag mAb (ABclonal) was used as the primary anti-body at a 1:4000 dilution, followed by incubation with a goat anti-rabbit IgG (HRP) secondary antibody (Abbkine). GAPDH protein was detected using GAPDH rabbit mAb antibody (ABclonal). Protein detection was performed using a LI-COR Odyssey Infrared Imaging System (Tanon 5200).

**Fig. S1.** (A) Total synthesis of *ent*-kaurene diterpenoids. (B) Reported plant-derived enzymes catalyze the selective oxidation at different positions of *ent*-kaurene diterpenoids.

**Fig. S2.** Transcriptome analysis of *I. amethystoides* tissues. (A) RNA extracted from the leaves, stems, and roots of *I. amethystoides* was used for RNA-Seq. (B) Overlap of gene annotations among NR (Non-Redundant Protein Database), NT (Nucleotide Database), KOG (Eukaryotic Orthologous Groups), GO (Gene Ontology), Pfam (Protein Families Database). (C) KEGG (Kyoto Encyclopedia of Genes and Genomes) pathway classification map. 22,025 genes were divided into five metabolic branches according to corresponding biological pathways, and 347 genes (1.58%) involved in terpenoid and polyketide metabolism.

**Fig. S3.** Sequence alignment of diTPSs in *I. amethystoides*. (A) Sequence alignment of six CPSs in *I. amethystoides* with “DXDD” motif. (B) Sequence alignment of five KSLs in *I. amethystoides* with “DDXXD” motif.

**Fig. S4.** Phylogenetic analysis and characterization of diterpenoid synthases. Phylogenetic tree of typical plant diterpenoid synthases with maximum-likelihood method. All the genes used here are listed in Table S3. PpCPS/KS was used as an outgroup, shown with grey shade. Six TPSs clades are shown with different color shadow. IamCPS1 and IamCPS5 are highlighted in red, IamKSL2 and IamKSL5 are highlighted in blue, the rest of IamCPSs and IamKSLs from *I. amethystoides* are indicated in green.

 **Fig. S5.** Revelation of co-expressed candidate CYPs based on *IamCPS1* and *IamCPS5*. (A) RNA-seq analysis combined with Pearson’s correlation using *IamCPS1* as bait gene. (B) RNA-seq analysis combined with Pearson’s correlation using *IamCPS5* as bait gene.

**Fig. S6.** Enzyme kinetics assays of IamCYP71D761 and IamCYP706V18. (A) Kinetic characterization of IamCYP71D761 for the conversion of *ent*-kaurene into **1**. The concentration of microsomal protein IamCYP71D761 is 40 nM. (B) Kinetic characterization of IamCYP71D761 for the conversion of **2** into **3**. (C) Kinetic characterization of IamCYP706V18 for the conversion of *ent*-kaurene into **2**. The concentration of microsomal protein IamCYP06V18 is 50 nM. (D) Kinetic characterization of IamCYP706V18 for the conversion of **1** into **3**. All enzymatic reactions were performed with 50 to 2000 μM substrate and a saturating concentration of cofactors at 30 °C for 1 h in a total volume of 300 μL containing ~3 pmol microsomal protein. *K_m_* and *k_cat_* are represented as mean values ± SD (n = 3 biologically independent replicates).

**Fig. S7.** Characterization of IamCYP71D761 and IamCYP706V18. (A) Conversion of **1** to **4** via PCC oxidation. (B) Conversion of **2** to **5** via PCC oxidation. (C) Microsome assays of IamCYP06V18 with **4**. (D) Microsome assays of IamCYP71D761 with substrate **5**. (E) Proposed biosynthetic pathway of typical *ent*-kaurene diterpenoids in *I. amethystoides*, composing GLA, GLB, wangzaozin A, and rabdosinatol.

**Fig. S8.** Western blot analysis of CEN.PK2-1D strains expressing *IamCYP71D761-GFP* and *IamCYP706V18-GFP*, with GAPDH used as a loading control.

**Fig. S9.** *In vitro* characterization of ScATF2. (A) SDS‑PAGE analysis of ScATF2 expressed from pET28a, with empty vector (EV) as a negative control. The target protein is marked with a red box. (B) GC‑MS analysis of compound **6** generated after incubation of ScATF2 with compound **3**.

**Fig. S10.** Production of *ent*-kaurenoids (*ent*-kaurene, **1**, **2**, and **3**) in engineered strains YC33–YC40. Data are presented as mean values ± SD (n = 3 biologically independent replicates).

**Fig. S11.** Phylogenetic tree of CYPs involved in *ent-*kaurene diterpenoids biosynthesis. The tree was constructed with maximum-likelihood method. All the genes used here are listed in Table S4. Genes identified in this work are shown with red color, genes from *I. amethystoides* are displayed with green color.

**Fig. S12.** NMR spectra of 3α-hydroxy-*ent*-kaurene (**1**). (A) Chemical formula. The ^1^H NMR data is shown in bule, and the ^13^C NMR data in red. (B) ^1^H NMR spectrum (CDCl_3_, 600 MHz). δ = 1.85 (1H, d, J = 13.3 Hz, H-1a), 0.90 (1H, d, J = 13.3 Hz, H-1b), 1.63 (2H, m, H-2), 3.20 (1H, m, H-3), 0.76 (1H, m, H-5), 1.57 (1H, m, H-6a), 1.38 (1H, m, H-6b), 1.51 (2H, m, H-7), 1.03 (1H, m, H-9), 1.63 (1H, m, H-11a), 1.55 (1H, m, H-11b), 1.64 (1H, m, H-12a), 1.48 (1H, m, H-12b), 2.64 (1H, s, H-13), 1.97 (1H, d, J = 11.3 Hz, H-14a), 1.11 (1H, d, J = 11.3 Hz, H-14b), 2.06 (2H, s, H-15), 4.79 (1H, s, H-17a), 4.47 (1H, s, H-17b), 0.98 (3H, s, H-18), 0.78 (3H, s, H-19), 1.02 (3H, s, H-20). (C) ^13^C NMR spectrum (CDCl_3_, 150 MHz). δ = 38.9 (C-1), 27.6 (C-2), 79.2 (C-3), 39.0 (C-4), 55.4 (C-5), 20.2 (C-6), 41.4 (C-7), 44.2 (C-8), 56.1 (C-9), 39.2 (C-10), 18.4 (C-11), 33.4 (C-12), 44.1 (C-13), 39.9 (C-14), 49.2 (C-15), 155.9 (C-16), 103.2 (C-17), 28.5 (C-18), 15.6 (C-19), 17.7 (C-20). 9.8 (C-16), 144.4 (C-17), 33.6 (C-18), 21.7 (C-19), 17.7 (C-20).

**Fig. S13.** NMR spectra of 15β-hydroxy-*ent*-kaurene (**2**). (A) Chemical formula. The ^1^H NMR data is shown in bule, and the ^13^C NMR data in red. (B) ^1^H NMR spectrum (CDCl_3_, 600 MHz). δ = 1.85 (1H, m, H-1a), 0.83 (1H, m, H-1b), 1.61 (2H, m, H-2), 1.37 (1H, m, H-3a), 1.15 (1H, m, H-3b), 0.81 (1H, m, H-5), 1.61 (1H, m, H-6a), 1.40 (1H, m, H-6b), 1.67 (1H, m, H-7a), 1.32 (1H, m, H-7b), 0.92 (1H, m, H-9), 1.61 (1H, m, H-11a), 1.40 (1H, m, H-11b), 1.66 (1H, m, H-12a), 1.46 (1H, m, H-12b), 2.66 (1H, br s, H-13), 2.01 (1H, d, J = 12.0 Hz, H-14a), 1.02 (1H, m, H-14b), 3.74 (1H, t, J = 12.0 Hz, H-15), 5.09 (1H, br s, H-17a), 4.96 (1H, s, H-17b), 0.86 (3H, s, H-18), 0.82 (3H, s, H-19), 1.04 (3H, s, H-20). (C) ^13^C NMR spectrum (CDCl_3_, 150 MHz). δ = 40.7 (C-1), 18.8 (C-2), 42.2 (C-3), 33.6 (C-4), 55.7 (C-5), 18.9 (C-6), 39.2 (C-7), 46.7 (C-8), 55.6 (C-9), 39.0 (C-10), 18.9 (C-11), 33.7 (C-12), 40.4 (C-13), 36.6 (C-14), 82.8 (C-15), 158.7 (C-16), 104.8 (C-17), 33.9 (C-18), 21.8 (C-19), 18.3 (C-20).

**Fig. S14.** NMR spectra of 3α,15β-dihydroxy-*ent-*kaurene (**3**). (A) Chemical formula. The ^1^H NMR data is shown in bule, and the ^13^C NMR data in red. (B) ^1^H NMR spectrum (CDCl_3_, 600 MHz). δ = 1.88 (1H, d, J = 12.9 Hz, H-1a), 0.98 (1H, m, H-1b), 1.62 (2H, m, H-2), 3.21 (1H, m, H-3), 0.77 (1H, m, H-5), 1.60 (1H, m, H-6a), 1.37 (1H, m, H-6b), 1.66 (1H, m, H-7a), 1.34 (1H, m, H-7b), 1.30 (1H, m, H-9), 1.55 (1H, m, H-11a), 1.44 (1H, m, H-11b), 1.65 (1H, m, H-12a), 1.47 (1H, m, H-12b), 2.66 (1H, s, H-13), 1.98 (1H, d, J = 12.0 Hz, H-14a), 1.02 (1H, m, H-14b), 3.75 (1H, s, H-15), 5.09 (1H, s, H-17a), 4.96 (1H, s, H-17b), 0.98 (3H, s, H-18), 0.78 (3H, s, H-19), 1.04 (3H, s, H-20). (C) ^13^C NMR spectrum (CDCl_3_, 150 MHz). δ = 38.8 (C-1), 27.5 (C-2), 79.1 (C-3), 38.8 (C-4), 54.6 (C-5), 19.8 (C-6), 38.9 (C-7), 45.7 (C-8), 46.5 (C-9), 38.9 (C-10), 18.3 (C-11), 33.4 (C-12), 40.3 (C-13), 36.6 (C-14), 82.5 (C-15), 158.5 (C-16), 105.0 (C-17), 28.5 (C-18), 15.6 (C-19), 17.7 (C-20).

**Fig. S15.** NMR spectra of 3-carbonyl-*ent*-kaurene (**4**). (A) Chemical formula. The ^1^H NMR data is shown in bule, and the ^13^C NMR data in red. (B) ^1^H NMR spectrum (CDCl_3_, 600 MHz). δ = 2.02 (1H, dt, J = 13.3, 6.3 Hz, H-1a), 1.40 (1H, m, H-1b), 2.47 (2H, dd, J = 8.6, 6.3 Hz, H-2), 1.43 (1H, overlap, H-5), 1.48 (2H, overlap, H-6), 1.52 (2H, m, H-7), 1.16 (1H, overlap, H-9), 1.71 (1H, m, H-11a), 1.53 (1H, m, H-11b), 1.64 (1H, tdd, J = 11.8, 4.7, 2.2 Hz, H-12a), 1.53 (1H, m, H-12b), 2.67 (1H, m, H-13), 1.95 (1H, dd, J = 11.8, 1.3 Hz, H-14a), 1.14 (1H, m, H-14b), 2.08 (2H, m, H-15), 4.82 (1H, s, H-17a), 4.76 (1H, s, H-17b), 1.08 (3H, s, H-18), 1.04 (3H, s, H-19), 1.08 (3H, s, H-20). (C) ^13^C NMR spectrum (CDCl_3_, 150 MHz). δ = 40.4 (C-1), 34.3 (C-2), 218.3 (C-3), 47.3 (C-4), 54.7 (C-5), 21.6 (C-6), 39.5 (C-7), 44.2 (C-8), 54.9 (C-9), 38.7 (C-10), 18.9 (C-11), 33.2 (C-12), 43.9 (C-13), 39.5 (C-14), 48.9 (C-15), 155.5 (C-16), 103.5 (C-17), 27.5 (C-18), 21.2 (C-19), 17.8 (C-20).

**Fig. S16.** NMR spectra of 15-carbonyl-*ent-*kaurene (**5**). (A) Chemical formula. The ^1^H NMR data is shown in bule, and the ^13^C NMR data in red. (B) ^1^H NMR spectrum (CDCl_3_, 600 MHz). δ = 1.78 (1H, dt, J = 13.1, 4.1 Hz, H-1a), 0.76 (1H, td, J = 13.1, 3.8, H-1b), 1.60 (1H, m, H-2a), 1.37 (1H, m, H-2b), 1.15 (1H, td, J = 14.1, 4.9 Hz, H-3a), 1.37 (1H, m, H-3b), 0.92 (1H, dd, J = 12.2, 2.4 Hz, H-5), 1.30 (1H, m, H-6a), 1.59 (1H, m, H-6b), 1.31 (1H, m, H-7a), 1.94 (1H, td, J = 13.5, 4.2 Hz, H-7b), 1.23 (1H, d, J = 8.8 Hz, H-9), 1.47 (1H, m, H-11a), 1.68 (1H, m, H-11b), 1.89 (1H, tdd, J = 13.3, 6.4, 2.5 Hz, H-12a), 1.67 (1H, m, H-12b), 3.03 (1H, m, H-13), 2.42 (1H, d, J = 11.8 Hz, H-14a), 1.37 (1H, m, H-14b), 5.93 (1H, s, H-17a), 5.24 (1H, s, H-17b), 0.88 (3H, s, H-18), 0.82 (3H, s, H-19), 1.09 (3H, s, H-20). (C) ^13^C NMR spectrum (CDCl_3_, 150 MHz). δ = 39.8 (C-1), 18.5 (C-2), 42.1 (C-3), 33.4 (C-4), 55.5 (C-5), 18.7 (C-6), 39.8 (C-7), 52.7 (C-8), 52.8 (C-9), 40.3 (C-10), 18.3 (C-11), 32.7 (C-12), 38.3 (C-13), 36.9 (C-14), 211.0 (C-15), 14

**Fig. S17.** NMR spectra of 3-acetoxy-15β-hydroxy-*ent-*kaurene (**6**). (A) Chemical formula. The ^1^H NMR data is shown in bule, and the ^13^C NMR data in red. (B) ^1^H NMR spectrum (CDCl_3_, 600 MHz). δ = 1.88 (1H, d, J = 13.1 Hz, H-1a), 1.05 (1H, m, H-1b), 1.65 (2H, m, H-2), 4.47 (1H, m, H-3), 0.86 (1H, m, H-5), 1.59 (1H, m, H-6a), 1.38 (1H, m, H-6b), 1.66 (1H, m, H-7a), 1.34 (1H, m, H-7b), 1.33 (1H, m, H-9), 1.54 (1H, m, H-11a), 1.45 (1H, m, H-11b), 1.64 (1H, m, H-12a), 1.47 (1H, m, H-12b), 2.66 (1H, s, H-13), 1.98 (1H, d, J = 12.0 Hz, H-14a), 1.03 (1H, m, H-14b), 3.75 (1H, s, H-15), 5.10 (1H, s, H-17a), 4.96 (1H, s, H-17b), 0.86 (3H, s, H-18), 0.86 (3H, s, H-19), 1.07 (3H, s, H-20), 2.04 (3H, s, H-22). (C) ^13^C NMR spectrum (CDCl_3_, 150 MHz). δ = 38.5 (C-1), 23.8 (C-2), 81.1 (C-3), 37.9 (C-4), 54.8 (C-5), 19.7 (C-6), 38.8 (C-7), 45.8 (C-8), 46.4 (C-9), 38.8 (C-10), 18.3 (C-11), 33.4 (C-12), 40.2 (C-13), 36.6 (C-14), 82.5 (C-15), 158.4 (C-16), 105.1 (C-17), 28.5 (C-18), 16.8 (C-19), 17.8 (C-20), 171.0 (C-21), 21.4 (C-22). **Table S1.** Plasmids used in this study.

| plasmid | description |
| --- | --- |
| pIRS | pCDFDuet bearing *dxs*, *dxr* and *idi* |
| pC1 | pACYCDuet bearing AgGGPS |
| pC2 | pACYCDuet bearing AgGGPS and ZmCPS2/An2 |
| pC3 | pACYCDuet bearing AgGGPS and IamCPS1 |
| pC4 | pACYCDuet bearing AgGGPS and IamCPS5 |
| pD1 | pET32a bearing AtKS |
| pD2 | pET32a PpCPS/KS |
| pD3 | pET32a PpCPS/KS:I741T |
| pD4 | pET32a bearing IamKSL2 |
| pD5 | pET32a bearing IamKSL5 |
| pATF2 | pET28a bearing ScATF2 |
| pESC-URA | multi-copy protein expression in *S. cerevisiae* host |
| pU1 | pESC-URA bearing XdGGPPS |
| pU2 | pESC-URA bearing GfKS |
| pU3 | pESC-URA bearing JsKS |
| pU4 | pESC-URA bearing PspKS |
| pU5 | pESC-URA bearing t85JsKS-MBP |
| pU6 | pESC-URA bearing t126JsKS-MBP |
| pU7 | pESC-URA bearing ATR1 |
| pU8 | pESC-URA bearing IamCYP71D761 |
| pU9 | pESC-URA bearing IamCYP706V18 |
| pU10 | pESC-URA bearing IamCYP71D761-GFP |
| pU11 | pESC-URA bearing IamCYP706V18-GFP |
| pRS415-LEU | mono-copy protein expression in *S. cerevisiae* host |
| pL1 | pRS415-LEU bearing promoter GAL1 to transcribe IamCYP71D761-optimized |
| pL2 | pRS415-LEU bearing promoter SptGAL2 to transcribe IamCYP71D761-optimized |
| pL3 | pRS415-LEU bearing promoter TEF1 to transcribe IamCYP71D761-optimized |
| pL4 | pRS415-LEU bearing promoter TDH3 to transcribe IamCYP71D761-optimized |
| pL5 | pRS415-LEU bearing promoter TDH3-RPS25Ai to transcribe IamCYP71D761-optimized |
| pL6 | pRS415-LEU bearing promoter eTDH3 to transcribe IamCYP71D761-optimized |
| pL7 | pRS415-LEU bearing promoter eTDH3-RPS25Ai to transcribe IamCYP71D761-optimized |
| pL8 | pRS415-LEU bearing promoter GAL1 to transcribe IamCYP706V18 |
| pL9 | pRS415-LEU bearing promoter SptGAL2 to transcribe IamCYP706V18 |
| pL10 | pRS415-LEU bearing promoter TEF1 to transcribe IamCYP706V18 |
| pL11 | pRS415-LEU bearing promoter TDH3 to transcribe IamCYP706V18 |
| pL12 | pRS415-LEU bearing promoter TDH3-RPS25Ai to transcribe IamCYP706V18 |
| pL13 | pRS415-LEU bearing promoter eTDH3 to transcribe IamCYP706V18 |
| pL14 | pRS415-LEU bearing promoter eTDH3-RPS25Ai to transcribe IamCYP706V18 |
| XII-1 gRNA | The gRNA was designed to target the XII-1 integration site |
| XII-4 gRNA | The gRNA was designed to target the XII-4 integration site |
| XII-5 gRNA | The gRNA was designed to target the XII-5 integration site |
| int14 gRNA | The gRNA was designed to target the int14 integration site |
| 1414a gRNA | The gRNA was designed to target the 1414a integration site |
| 308a gRNA | The gRNA was designed to target the 308a integration site |
| 106a gRNA | The gRNA was designed to target the 106a integration site |
| X-2 gRNA | The gRNA was designed to target the X-2 integration site |
| 1021b gRNA | The gRNA was designed to target the 1021b integration site |
| XI-6 gRNA | The gRNA was designed to target the XI-6 integration site |
| IME4p gRNA | The gRNA was designed to target the *IME4*p integration site |
| ATF1 gRNA | The gRNA was designed to target the *ATF1* integration site |
| ATF2 gRNA | The gRNA was designed to target the *ATF2* integration site |

**Table S2.** Primers used in this study.

| Primers used for construction of plasmids | |
| --- | --- |
| 2F-DuetUP2 | TTGTACACGGCCGCATAATC |
| T7-ter | TGCTAGTTATTGCTCAGCGG |
| S-Tag | CGAACGCCAGCACATGGACA |
| IamCPS1-pACYC-F | CATCATCACCACAGCCAGATGAATCAAGAGGTGGTATCA |
| IamCPS1-pACYC-R | GCGCCGAGCTCGAATTCGCTAATCTATTTTTTCGAACAAC |
| IamCPS5-pACYC-F | CCATCATCACCACAGCCAGATGGTACGAGTTAATTCAAGCT |
| IamCPS5-pACYC-R | CGCCGAGCTCGAATTCGCTAATCTACGTTTTCGAACAGTA |
| IamKSL2-32a-F | GCCATGGCTGATATCGATGTCTCTTATTCTCTCCTCTTTCTC |
| IamKSL2-32a-R | GACGGAGCTCGAATTCGTTAGAAGTGGTTGAGGAGAATAGGT |
| IamKSL5-32a-F | the same as IamKSL2-32a-F |
| IamKSL5-32a-R | the same as IamKSL2-32a-R |
| PpCPS-32a-F | GCCATGGCTGATATCGATGGCTTCCTCTACCTTGATC |
| PpCPS-32a-R | GACGGAGCTCGAATTCGTCATTCAGGAACTGGTTCA |
| PpI741T-F | TGCTTTGGAACCTACCGTTTGCTCCACT |
| PpI741T-R | AGTGGAGCAAACGATGGGTTCCAAAGCA |
| XdGGPPS-GAL1-F | AGGAGAAAAAACCCCGAAAAAAATGGATTACGCTAACATTTTGA |
| XdGGPPS-GAL1-R | TATAGTGAGTCGTATTACGTTATAATGGAATATCAGCCAACTTC |
| GfKS-GAL1-F | AGGAGAAAAAACCCCGAAAAAAATGCCGGGTAAGATTGAAAAC |
| GfKS-GAL1-R | TATAGTGAGTCGTATTACGCTACTTCATGCTGGAGGACAAATC |
| JsKS-GAL1-F | AGGAGAAAAAACCCCGAAAAAAATGGTTCCACCATTACAAGC |
| JsKS-GAL1-R | TATAGTGAGTCGTATTACGTCAGACAACTGGCTGGAACA |
| PspKS-GAL1-F | AGGAGAAAAAACCCCGAAAAAAATGTTCGCCAAGTTCGATATG |
| PspKS-GAL1-R | TATAGTGAGTCGTATTACGTCAAGTGCCAACGTGCTTCA |
| pESC-Cter-MBP-R | ATAGTGAGTCGTATTACGTTATTTGGTAATTCTTGTC |
| pESC-Cter-MBP-F | GGTGGTGGTGGCGGTGGTTCAAAGATCGAGGAAGGA |
| JsKS-MBP-R | AACCACCGCCACCACCACCGACAACTGGCTGGAACAAAAC |
| t126JsKS-MBP-F | AGGAGAAAAAACCCCGAAAAAAATGTACAAGGACAAGGGTAAAG |
| t85JsKS-MBP-F | AGGAGAAAAAACCCCGAAAAAAATGGTTCCACCATTACAAGC |
| GAL1-ATR1-F | ACGTCAAGGAGAAAAAACCCCAAAAAAATGACTTCTGCCTTGTATGCCTCT |
| GAL1-ATR1-R | TATAGTGAGTCGTATTACGTCACCAGACATCTCTCAAGTATC |
| GAL1-IamCYP71D761-F | ACGTCAAGGAGAAAAAACCCCAAAAAAATGCTCACACTCATTTTCTCTAC |
| GAL1-IamCYP71D761-R | TATAGTGAGTCGTATTACGTTAACGAGCCACAAAAGGCCTCT |
| GAL1-IamCYP706V18-F | ACGTCAAGGAGAAAAAACCCCAAAAAAATGAAAGCGGCGTTACTAATG |
| GAL1-IamCYP706V18-R | TATAGTGAGTCGTATTACGCTAGTTATATAGCTCCGGATGCGG |
| pRS415-F1 | GGATCCGTAATACGACTCACTATAGGGCCCGGGC |
| pRS415-R1 | GGATCCCCCGGGCTGCAGGAATTCGATA |
| GAL1-F2 | CTGCAGCCCGGGGGATCCCGGATTAGAAGCCGCCGA |
| SptGAL2-F2 | CTGCAGCCCGGGGGATCCACCCCACCAGATCCGGA |
| TEF1-F2 | CTGCAGCCCGGGGGATCC |
| TDH3-F2 | CTGCAGCCCGGGGGATCCTCGAGTTTATCATTATCA |
| eTDH3-F2 | CTGCAGCCCGGGGGATCCTGTTTCTACTCCTTTTTTACTC |
| GAL1-R2 | CTCCTTGACGTTAAAGTATAGAGG |
| SptGAL2-R2 | TTTGAATGTGTATATATAATATTATTTGTA |
| TEF1-R2 | TTTGTAATTAAAACTTAGATTAGATTGCTATGC |
| TDH3-R2 | TTTGTTTATGTGTGTTTATTCGAAAC |
| TDH3-Ai-R2 | GATTTATTAAATGTTCTATGATAAAGTAA |
| GAL1-IamCYP71D761y-F3 | CTATACTTTAACGTCAAGGAGAAAAAAATGTTGACCTTGATTTTCTCT |
| SptGAL2-IamCYP71D761y-F3 | ATATTATATATACACATTCAAAAAAAAAATGTTGACCTTGATTTTCTCT |
| TEF1-IamCYP71D761y-F3 | ATCTAATCTAAGTTTTAATTACAAAAAAAAAATGTTGACCTTGATTTTCTCT |
| TDH3-IamCYP71D761y-F3 | GAATAAACACACATAAACAAAAAAAAAATGTTGACCTTGATTTTCTCT |
| TDH3-Ai-IamCYP71D761y-F3 | TATCATAGAACATTTAATAAATCAAAAAAATGTTGACCTTGATTTTCTCT |
| IamCYP71D761y-415-R3 | CGGGCCCTATAGTGAGTCGTATTACGGATCCTCAACGAGCAACGAATGGT |
| GAL1-IamCYP706V18-F | CTATACTTTAACGTCAAGGAGAAAAAAATGAAAGCGGCGTTACTAAT |
| SptGAL2-IamCYP706V18-F | ATATTATATATACACATTCAAAAAAAAAATGAAAGCGGCGTTACTAAT |
| TEF1-IamCYP706V18-F | ATCTAATCTAAGTTTTAATTACAAAAAAAAAATGAAAGCGGCGTTACTAAT |
| TDH3-IamCYP706V18-F | GAATAAACACACATAAACAAAAAAAAAATGAAAGCGGCGTTACTAAT |
| TDH3-Ai-IamCYP706V18-F | TATCATAGAACATTTAATAAATCAAAAAAATGAAAGCGGCGTTACTAAT |
| IamCYP706V18-415-R3 | GCCCTATAGTGAGTCGTATTACGGATCCCTAGTTATATAGCTCCGGATGCG |
| GAL10-AaCYB5-F | CTCACTAAAGGGCGGCCGCCCACAAAAAAATGGCTTCAGATCCTAAAAACTTC |
| GAL10-AaCYB5-R | ATCGATACTAGTGCGGCCGCTTAAGCGCTTCTCTCCTTCGT |
| GAL10-CrCYB5-F | CTCACTAAAGGGCGGCCGCCCACAAAAAAATGGCTTCCGACCAAAAGTT |
| GAL10-CrCYB5-R | ATCGATACTAGTGCGGCCGCTCACTTTTCCTTGGTGTAATG |
| GAL10-RsCYB5-F | CTCACTAAAGGGCGGCCGCCCACAAAAAAATGGCCAGCGATCCGAAAG |
| GAL10-RsCYB5-R | ATCGATACTAGTGCGGCCGCTTATTCTTTTTTGGTGAAGTGGC |
| GAL10-TwCYB5-F | CTCACTAAAGGGCGGCCGCCCACAAAAAAATGGCATCCGACAGGAAGAT |
| GAL10-TwCYB5-R | ATCGATACTAGTGCGGCCGCTCACTCCTTCTTGGTGAAGTG |
| Primers used for gene-edition of *Saccharomyces cerevisiae* | |
| gRNA-P1 | GCGGTTAGCTCCTTCGGTCCTCCGATCGTTGTCAGAAGTAAGTTGGCCGCAGTGTTATC |
| gRNA-P2 | GATAACACTGCGGCCAACTTACTTCTGACAACGATCGGAGGACCGAAGGAGCTAACCGC |
| gRNA-IME4p-1L | AAACTTCTCCGCAGTGAAAGATAAATGATCTCCCTTTCCTATAGCTCCAGGTTTTAGAGCTAGAAATAG |
| gRNA-IME4p-2L | AAACTTCTCCGCAGTGAAAGATAAATGATCTACCGATCTTCCAGAATGCCGTTTTAGAGCTAGAAATAG |
| IME4p-GAL1-F | ATCACGTTTAACGATGCCCCACGGATTAGAAGCCGCCGA |
| IME4p-GAL1-R | GTACTAGTTTATCGTTAATCATCTCCTTGACGTTAAAGTATAG |
| IME4p-SptGAL2-F | ATCACGTTTAACGATGCCCCAACCCCACCAGATCCGGA |
| IME4p-SptGAL2-R | GTACTAGTTTATCGTTAATCATTTTGAATGTGTATATATAATAT |
| IME4p-TEF2-F | ATCACGTTTAACGATGCCCCATATACTTACATATAGTAGATGTC |
| IME4p-TEF2-R | GTACTAGTTTATCGTTAATCATGGTACTAGTGTTTAGTTAATTATAG |
| IME4p-PGK1-F | ATCACGTTTAACGATGCCCCAACGCACAGATATTATAACATC |
| IME4p-PGK1-R | GTACTAGTTTATCGTTAATCATTGTTTTATATTTGTTGTAAAAAGTAG |
| IME4p-TDH3-F | ATATCACGTTTAACGATGCCCCATCGAGTTTATCATTATCA |
| IME4p-TDH3-R | GTACTAGTTTATCGTTAATCATTTTGTTTATGTGTGTTTATTCG |
| IME4p-TDH3-Ai-R | GTACTAGTTTATCGTTAATCATGATTTATTAAATGTTCTATG |
| IME4p-up-F | TCAAGTGTGCCTCTGGGCAGTTTGT |
| IME4p-up-R | TGGGGCATCGTTAAACGTGATATTCCT |
| IME4p-down-F | ATGATTAACGATAAACTAGTACATTTTCTGATCC |
| IME4p-down-R | TCAATAAATTTTGTAAGCGATTGCTCAG |
| IME4p-test-F | GTCAAGGCTCGCACCAGTGT |
| IME4p-test-R | GACAATGTACACCGAGGTGGAC |
| gRNA-ScATF1-1L | AAACTTCTCCGCAGTGAAAGATAAATGATCTTCGGAATAAACAAGTACGGGTTTTAGAGCTAGAAATAG |
| gRNA-ScATF1-2L | AAACTTCTCCGCAGTGAAAGATAAATGATCAAAATATGTGAACATTGACAGTTTTAGAGCTAGAAATAG |
| gRNA-ScATF2-1L | AAACTTCTCCGCAGTGAAAGATAAATGATCGTTTATGTCACTAACCACTGGTTTTAGAGCTAGAAATAG |
| gRNA-ScATF2-2L | AAACTTCTCCGCAGTGAAAGATAAATGATCGGCCTCTTCGGATTACCCAGGTTTTAGAGCTAGAAATAG |
| ATF1-up-F | TCATTTGTTGGCACAGGACT |
| ATF1-up-R | AGTCAAGCATCATGTGAGATCATGAGAGCTGATAAATTGATGGTA |
| ATF1-down-F | ATCTCACATGATGCTTGACT |
| ATF1-down-R | TATACCAGCTCCCACAGGAC |
| ATF1-test-F | ACTGACACCCGGATAATTAAG |
| ATF1-test-R | GTTTCTTGGGTGGGAGAAGT |
| ATF2-up-F | CAGGCTACATTGAACTCTGT |
| ATF2-up-R | GCTACGGCAGTATCGCATTAATCTTCCATAATTAACCTGGACAAT |
| ATF2-down-F | TAATGCGATACTGCCGTAGC |
| ATF2-down-R | CAGACATGACCCTTTTCTCG |
| ATF2-test-F | TGAACTGGAAGGCTCATCCA |
| ATF2-test-R | GTAGCAACACATGGACCGAT |
| XII-5-GAL1-F | GAAAACTAAATCTCAATGATTAACTCCATTTAATCCTTTTTTGAAAGGCAAAAGAGGTCCCCGGATTAGAAGCCGCCGA |
| XII-5-CYC1-R | CTTTTTATATGTTTTTACTTCTGTATATCGTGAAGTAGTAAGTGATAAGCGAATTTGGCTACTTCGAGCGTCCCAAAACCT |
| XII-5-test-F | ATTTCTCACGGTAGAGCGGA |
| XII-5-test-R | TTTGATACTGTGGTTGCTGCA |
| XII-1-GAL1-F | GGCAAATCCCAGATTTGGCTTTGATTTTGGCATCGGTTCGGTTCTTTCAAATATCTTCTTCCCCGGATTAGAAGCCGCCGA |
| XII-1-CYC1-R | GCTTGTAAAATTTATTATTACAGTCACTCAGACAGAGCACTAAACTGAACTAGTTATTAACTTCGAGCGTCCCAAAACCT |
| XII-1-test-F | TTGGACCACTTCTTCTTGGAC |
| XII-1-test-R | CAGCGTGACAATAATGAGCAT |
| XII-4-TDH3-F | CAGCGATAGTTTTTTTCCATCACACGTACTATGGCACCAACTTTGTACTATTCCTTCCCCTCGAGTTTATCATTATCAAT |
| XII-4-CYC1-R | GGTAACATACCTCCGTGAGGCATCCTTTTATTTGACTCTAATGGGGAATTGTACAAATTGAAAACCTTCTCAAGCAAGGT |
| XII-4-test-F | GCTTCAAGAATTGAGTAAAC |
| XII-4-test-R | GCTTCAAGAATTGAGTAAAC |
| 308a-TDH3-F | CAGCGTAGCTAAACTCTAAAATTTATCTAAATCACTCATATAAACCGAACCCTTCCCCTTCTCGAGTTTATCATTATCAATACTG |
| 308a-CYC1-R1 | TCTGTGGATTTTCCATATTGTTTGGCGCTGATCGCCATGCCATGTTTATGAAATGTATAGAAAACCTTCTCAAGCAAGGT |
| 308a-test-F | CAGGGAATCGATGAGGTTGT |
| 308a-test-R | TGAATGGGGCGGAAATTCAT |
| int14-GAL1-F | AATCTCGCAATCGGAAAAACACTCGCGAGAACCAAAACAAGGATCCCCTAAACCCAGTACCGGATTAGAAGCCGCCGA |
| int14-CYC1-R | GAAGCGATCCGTAGTCCTATCCGGGTAACACTCATCGTCCGGCCTCCGCCCCCTTTTCCCCTTCGAGCGTCCCAAAACCT |
| int14-test-F | ACTGGGTTTAGGGGATCCTTG |
| int14-test-R | TCTCCCGGGGAAAAGGGGGC |
| 1414a-eTDH3-F | TGTTCTTTTTCTTTTTTTGGAGATGCGTCTTAAAAAGCGCCACAGTTTCAAGGGTCCGGTGTTTCTACTCCTTTTTTACTC |
| 1414a-CYC1-R | TGGTTCATTCGCAAATTCATGAAGTATTATTTAATACACAGTAATCTGGCATTTCATACTACTTCGAGCGTCCCAAAACCTTCTCA |
| 1414a-test-F | AGGTTCATTGGTTGCTCAAT |
| 1414a-test-R | TGGTTGTTCCATGCAGGAGT |
| 106a-eTDH3-F | TATTTTTTTCTTCCTTATTATTTTTTTAGTTCTGAAGTTTGACCAGGGCGCTACCCTGACCGACGACTCACTATAGGGCGA |
| 106a-CYC1-R | ATGTAAATAAACAATCACAAGGAAAAAAAAAAAAAAAAAAAAAATAGCCGCCATGACCCCGAAAACCTTCTCAAGCAAGGT |
| 106a-test-F | CAACAGCAAAAAAGAGGGTC |
| 106a-test-R | ATATCGTTTTGATGGCGAGC |
| X-2-GAL10-F | CTCACTGAAAAAAGAAACGAGCGGAGGAATAGTATGATAAATCTTCAGCATAGATGGGTAACTACTTCAATATAGCAATGAGCAG |
| X-2-CYC1-R | CTCACTGAAAAAAGAAACGAGCGGAGGAATAGTATGATAAATCTTCAGCATAGATGGGTAACCTTCGAGCGTCCCAAAACCT |
| X-2-test-F | TGCTACTGGCAACTGTTG |
| X-2-test-R | AGACGCCACCAAGACAAT |

**Table S3.** Accession numbers for the diTPSs used in the phylogenetic analyses in Fig. 2D, S4.

| Protein | GenBank | TPS-clade |
| --- | --- | --- |
| AtKS | AAC39443 | e |
| AtTPS1 | AT4G15870.1 | a |
| AtTPS2 | AT4G16730.1 | b |
| AtTPS3 | AT4G16740.1 | b |
| AtTPS4 | AT1G61120.1 | f |
| AtTPS7 | AT4G20200.1 | a |
| AtTPS8 | AT4G20210.1 | a |
| AtTPS21 | AT5G23960.1 | g |
| IadKSL3 | PQ642451 | e |
| IadKSL5 | PQ642452 | e |
| IeCPS1 | AEP03177 | c |
| IeCPS2 | AEP03175 | c |
| IerKSL4 | PQ642453 | e |
| IerKSL5 | PQ642454 | e |
| IerKSL6 | PQ642455 | e |
| IpaCPS1 | PQ642445 | c |
| IpaCPS2 | PQ642446 | c |
| IpaKSL1 | PQ642447 | e |
| IpaKSL2 | PQ642448 | e |
| IpaKSL3 | PQ642449 | e |
| IpaKSL4 | PQ642450 | e |
| IrCPS1 | KU180499 | c |
| IrCPS2 | KU180500 | c |
| IrCPS3 | KU180501 | c |
| IrCPS4 | KU180502 | c |
| IrCPS5 | KU180503 | c |
| IrKSL1 | KU180504 | e |
| IrKSL2 | KU180505 | e |
| IrKSL3 | KU180506 | e |
| IrKSL4 | KX580633 | e |
| IrKSL5 | KX580634 | e |
| IrKSL6 | KX580635 | e |
| LjTPS1 | MZ557361 | c |
| LjTPS3 | MZ557362 | c |
| LjTPS4 | MZ557363 | c |
| LjTPS5 | MZ557364 | e |
| LjTPS6 | MZ557365 | e |
| LjTPS7 | MZ557366 | f |
| OsCPS1 | BAD42449 | c |
| OsKSL1 | AAQ72559 | e |
| OsKSL5 | Q6Z5J6 | e |
| OsKSL7 | BAC56714 | e |
| SmCPS1 | KC814639 | c |
| SmCPS2 | KC814640 | c |
| SmCPS4 | KP063138 | c |
| SmCPS5 | KC814642 | c |
| SmKSL1 | ABV08817 | e |
| SmKSL2 | KC814643 | e |
| TwTPS27 | KU948698 | b |
| TwTPS9 | KU948709 | c |
| ZmKSL3 | DAA36069 | e |
| ZmKSL4 | DAA49845 | e |
| ZmTPS1 | AAO18435 | e |
| ZmCPS2/An2 | NM_001111787.2 | c |
| PpCPS/KS | BAF61135 | outgroup |

**Table S4.** Accession numbers for the plant CYPs used in the phylogenetic analyses in Fig. S13.

| Protein | GenBank | Source |
| --- | --- | --- |
| IrCYP706V2 | UVC58033 | [1] |
| IrCYP706V3 | UVC58034 |  |
| IrCYP706V8 | UVC58039 |  |
| IrCYP706V6 | UVC58037 |  |
| IrCYP706V7 | UVC58038 |  |
| AtKAO1 | AF318500 | [2] |
| OsKAO | Q5VRM7 |  |
| OsKO2 | Q5Z5R4 |  |
| SmKO | KJ606394 |  |
| SrKO1 | AY364317 |  |
| AtCYP714A1 | AT5G24910 | [3] |
| OsCYP701A8 | Q0DBF4 | [4] |
| SpCYP71BE52 | KT157042 | [5] |
| TwCYP71BE85 | ON376001 | [6] |
| TwCYP71BE86 | ON375999 |  |
| TwCYP71BE271 | XP_038686650.1 | [7] |
| CfCYP71D381 | KT382342 | [2] |
| MvCYP71AU87 | MH593587 | [8] |
| TwCYP728B70 | TW009876.1 | [9] |
| OsCYP71Z6 | A3A871 | [10] |
| ZmCYP71Z16 | AQK65314.1 | [11] |
| IlCYP76AH42 | OP891585 | [12] |
| CfCYP76AH11 | KT382349 | [13] |
| CfCYP76AH16 | KT382359 |  |
| SmCYP76AH1 | JX422213 |  |
| SmCYP76AK1 | KR140169 | [5] |
| SmCYP76AK2 | KP337688.1 |  |
| VacCYP76BK1 | MG696754 | [14] |
| OsCYP76M8 | Q6YTF1 | [15] |
| OsCYP99A2 | NP_001406006 | [16] |
| OsCYP99A3 | Q0JF01 |  |
| PcCYP720B1 | KJ845671 | [2] |
| GbCYP7005C1 | UTS77781 | [17] |
| CpCYP964A1 | LC494434 | [18] |
| TwCYP72D19 | XM_038854645 | [6] |
| TwCYP81AM1 | WEW62272 |  |
| TwCYP82D274 | ON376001 | [19] |
| TwCYP82D263 | XP_038716720.1 |  |
| AcoCYP725A1 | / | [20] |
| AcoCYP72A1 | / |  |
| AcoCYP716B1 | / |  |
| AcoCYP71A1 | / |  |
| AcoCYP82C1 | / |  |
| AcoCYP93A1 | / |  |
| AcaCYP72A1 | / |  |
| AcaCYP71A1 | / |  |
| AcaCYP82C1 | / |  |
| AcaCYP93A1 | / |  |
| EfCYP82J33 |  | [21] |
| EfCYP71BF25 |  |  |
| EfCYP71D450 |  | [22] |

**References**

[1] Sun Y, Shao J, Liu H, Wang H, Wang G, Li J, et al. A chromosome-level genome assembly reveals that tandem-duplicated CYP706V oxidase genes control oridonin biosynthesis in the shoot apex of *Isodon rubescens*. Mol Plant 2023;16(3):517-532. <https://doi.org/10.1016/j.molp.2022.12.007>.
[2] Bathe U, Tissier A. Cytochrome P450 enzymes: A driving force of plant diterpene diversity. Phytochemistry 2019;161:149-162. <https://doi.org/10.1016/j.phytochem.2018.12.003>.
[3] Nomura T, Magome H, Hanada A, Takeda-Kamiya N, Mander LN, Kamiya Y, et al. Functional Analysis of *Arabidopsis* CYP714A1 and CYP714A2 Reveals That They are Distinct Gibberellin Modification Enzymes. Plant Cell Physiol 2013;54(11):1837-1851. <https://doi.org/10.1093/pcp/pct125>.
[4] Wang Q, Hillwig ML, Wu Y, Peters RJ. CYP701A8: a rice *ent*-kaurene oxidase paralog diverted to more specialized diterpenoid metabolism. Plant Physiol 2012;158(3):1418-1425. <https://doi.org/10.1104/pp.111.187518>.
[5] Banerjee A, Hamberger B. P450s controlling metabolic bifurcations in plant terpene specialized metabolism. Phytochem Rev 2018;17 (1):81-111. <https://doi.org/10.1007/s11101-017-9530-4>.
[6] Gao J, Ma L, Liu Y, Tu L, Wu X, Wang J, et al. CYP72D19 from *Tripterygium wilfordii* catalyzes C-2 hydroxylation of abietane-type diterpenoids. Plant Cell Rep 2023;42 (11):1733-1744. <https://doi.org/10.1007/s00299-023-03059-w>.
[7] Wang J, Xie Q, Wang X, Long M, Chen Y, Liu Z, et al. Discovery of Key Cytochrome P450 Monooxygenase (C20ox) Enables the Complete Synthesis of Tripterifordin and Neotripterifordin. ACS Catal 2025;15(3):2690-2702. <https://doi.org/10.1021/acscatal.4c07121>.
[8] Karunanithi, P. S.; Dhanota, P.; Addison, J. B.; Tong, S.; Fiehn, O.; Zerbe, P. Functional characterization of the cytochrome P450 monooxygenase CYP71AU87 indicates a role in marrubiin biosynthesis in the medicinal plant *Marrubium vulgare*. BMC Plant Biolo 2019;19:114.<https://doi.org/10.1186/s12870-019-1702-5>.
[9] Tu L, Su P, Zhang Z, Gao L, Wang J, Hu T, et al. Genome of Tripterygium wilfordii and identification of cytochrome P450 involved in triptolide biosynthesis. Nat Commun 2020;11(1):971. <https://doi.org/10.1038/s41467-020-14776-1>.
[10] Wu Y, Hillwig ML, Wang Q, Peters RJ. Parsing a multifunctional biosynthetic gene cluster from rice: Biochemical characterization of CYP71Z6 & 7. FEBS Lett 2011;585(21):3446-3451. <https://doi.org/10.1016/j.febslet.2011.09.038>.
[11] Ding Y, Murphy KM, Poretsky E, Mafu S, Yang B, Char SN, et al. Multiple genes recruited from hormone pathways partition maize diterpenoid defences. Nat Plants 2019;5(10):1043-1056. <https://doi.org/10.1038/s41477-019-0509-6>.
[12] Du Z, Peng Z, Yang H, Wu H, Sun J,Huang L. Identification and functional characterization of three cytochrome P450 genes for the abietane diterpenoid biosynthesis in *Isodon lophanthoides*. Planta 2023;257(5):90. <https://doi.org/10.1007/s00425-023-04125-z>.
[13] Pateraki I, Andersen-Ranberg J, Jensen NB, Wubshet SG, Heskes AM, Forman V, et al. Total biosynthesis of the cyclic AMP booster forskolin from *Coleus forskohlii*. Elife 2017;6. <https://doi.org/10.7554/eLife.23001>.
[14] Heskes AM, Sundram TCM, Boughton BA, Jensen NB, Hansen NL, Crocoll C, et al. Biosynthesis of bioactive diterpenoids in the medicinal plant *Vitex agnus-castus*. Plant J 2018;93(5):943-958. <https://doi.org/10.1111/tpj.13822>.
[15] Wang Q, Hillwig ML, Okada K, Yamazaki K, Wu Y, Swaminathan S, et al. Characterization of CYP76M5-8 indicates metabolic plasticity within a plant biosynthetic gene cluster. J Biol Chem 2012;287(9):6159-6168. <https://doi.org/10.1074/jbc.M111.305599>.
[16] De La Pena R,Sattely ES. Rerouting plant terpene biosynthesis enables momilactone pathway elucidation. Nat Chem Biol 2021;17(2):205-212. <https://doi.org/10.1038/s41589-020-00669-3>.
[17] Forman V, Luo D, Geu-Flores F, Lemcke R, Nelson DR, Kampranis SC, et al. A gene cluster in *Ginkgo biloba* encodes unique multifunctional cytochrome P450s that initiate ginkgolide biosynthesis. Nat Commun 2022;13(1):5143. <https://doi.org/10.1038/s41467-022-32879-9>.
[18] Mao L, Kawaide H, Higuchi T, Chen M, Miyamoto K, Hirata Y, et al. Genomic evidence for convergent evolution of gene clusters for momilactone biosynthesis in land plants. Proc Natl Acad Sci U S A 2020;117(22):12472-12480. <https://doi.org/10.1073/pnas.1914373117>.
[19] Zhang Y, Gao J, Ma L, Tu L, Hu T, Wu X, et al. Tandemly duplicated CYP82Ds catalyze 14-hydroxylation in triptolide biosynthesis and precursor production in *Saccharomyces cerevisiae*. Nat Commun 2023;14(1):875. <https://doi.org/10.1038/s41467-023-36353-y>.
[20] Luo F, Zhou Q, Chen F, Liu X, Chiu TY, Zhu GY, et al. Divergent multifunctional P450s-empowered biosynthesis of bioactive tripterifordin and cryptic atiserenoids in *Aconitum* implies convergent evolution. Nat Commun 2025;16(1):5857. <https://doi.org/10.1038/s41467-025-61188-0>.
[21] Lu K, Zhang R, Gao K, Li N, Cai Z, Zhu J, et al. Machine-Learning-Guided Discovery of Cytochrome P450 Enzymes for Bioproduction of Jolkinolides and Other Labdane-Related Diterpenoids. J Am Chem Soc 2026;148 (18):19369-19380. <https://doi.org//10.1021/jacs.6c05010>.
[22] Gao K, Zhao L, Wang L, Zhang R, Zhu J, Lin P, et al. Mining substrate-promiscuity cytochrome P450s from *Euphorbia Fischeriana* for heterologous bioproduction of diverse Labdane-Related Diterpenoids. Chin Che Let 2026;37(1):110903. <https://doi.org/10.1016/j.cclet.2025.110903>.
